# Peripandemic outcomes of infants treated for sentinel congenital heart diseases in England and Wales

**DOI:** 10.1136/openhrt-2024-002964

**Published:** 2025-02-17

**Authors:** Qi Huang, Deborah A Lawlor, John Nolan, Ferran Espuny-Pujol, Massimo Caputo, Christina Pagel, Sonya Crowe, Rodney CG Franklin, Kate L Brown, CVD-COVID-UK/COVID-IMPACT Consortium

**Affiliations:** 1 Clinical Operational Research Unit, Department of Mathematics, University College London, London, UK; 2 Population Health Science,Bristol Medical School, University of Bristol, Bristol, UK; 3 Medical Research Council Integrative Epidemiology, University of Bristol, Bristol, UK; 4 British Heart Foundation Data Science Centre, Health Data Research UK, London, UK; 5 Department of Computer Science, University of Reading, Reading, UK; 6 Cardiac Surgery, Translational Health Sciences, University of Bristol, Bristol, UK; 7 Paediatric Cardiology, Royal Brompton & Harefield NHS Foundation Trust, London, UK; 8 Cardiorespiratory, NIHR Great Ormond Street Hospital Biomedical Research Centre, London, UK; 9 Institute of Cardiovascular Science, University College London , London, UK

**Keywords:** Heart Defects, Congenital, COVID-19, Epidemiology, Risk Factors

## Abstract

**Background:**

Infants with congenital heart disease (CHD) are clinically vulnerable to cardiac deteriorations and intercurrent infections. We aimed to quantify the impact of health system disruptions during the COVID-19 pandemic, on their clinical outcomes and whether these differed by socioeconomic and ethnic subgroups.

**Methods:**

In this population-based cohort study, we used linked electronic healthcare datasets from England and Wales to identify infants with nine sentinel CHDs born and undergoing intervention in 2018–2022. The outcomes of cardiac intervention timing, infant mortality and hospital care utilisation, were described by birth eras, and risk factors were explored using multivariable regression.

**Results:**

Of 4900 included infants, 1545 (31.5%) were born prepandemic (reference), 1175 (24.0%) in the transition period, 1375 (28.0%) during restrictions and 810 (16.5%) postrestrictions. The casemix was hypoplastic left heart syndrome (195; 3.9%), functionally univentricular heart (180; 3.7%), transposition (610; 13.5%), pulmonary atresia (290; 5.9%), atrioventricular septal defect (590; 12.1%), tetralogy of Fallot (820; 16.7%), aortic stenosis (225; 4.6%), coarctation (740; 15.1%) and ventricular septal defect (1200; 24.5%).

Compared with prepandemic, there was no evidence for delay in treatment procedures in transition, restrictions or postrestrictions eras. Infant mortality increased for those born in the transition period, adjusted OR 1.60 (95% CI 1.06, 2.42) p=0.01, but not in restrictions or postrestrictions. The days spent at home were similar with birth in transition and restrictions, but fewer for postrestrictions, adjusted days difference −2 (95% CI −4, 0), p=0.05.

Outcomes did not vary by pandemic birth era according to social characteristics. There was higher infant mortality in the deprived versus non-deprived binary category (adjusted OR 1.56 (95% CI 1.11, 2.18), p=0.004) and there were fewer days spent at home for the most versus least deprived neighbourhood quintile (adjusted difference −4 (95% CI −6, –2), p<0.001).

**Conclusions:**

Specialist care for infants with CHD during the pandemic, in terms of pathway procedure timing and healthcare contacts, was not compromised. Increased healthcare utilisation postpandemic and heath inequality based on socioeconomic status require further evaluation.

**WHAT IS ALREADY KNOWN ON THIS TOPIC**

- Infancy is the highest risk period of life for those with congenital heart diseases (CHDs).
- Complex CHD is associated with serial planned surgeries in infancy and the requirement for careful monitoring and healthcare interventions when deterioration occurs.
- Healthcare services were affected by the COVID-19 pandemic.

**WHAT THIS STUDY ADDS**

- For infants with sentinel CHDs born in England and Wales, the consistency of age distributions at time of surgery for those born in the restriction and postrestriction periods compared with those born prepandemic, indicates no delays by pandemic-related health service disruptions.
- Infants who were born after the pandemic started had similar mortality to those who were infants before the pandemic.
- Infants who were born during pandemic restrictions and then especially those born postrestrictions had greater hospital care utilisation than those born before the pandemic.
- Pathway procedure timing, infant mortality and hospital care utilisation did not differ between ‘pandemic’ birth eras based on social characteristics. Across all birth eras combined, there was evidence that deprivation was associated with higher infant mortality and inpatient care utilisation.

**HOW THIS STUDY MIGHT AFFECT RESEARCH, PRACTICE OR POLICY**

- The increased hospital inpatient stays among infants with CHD born after restrictions ended require further exploration.
- Observed links between neighbourhood deprivation and outcomes require further exploration and could inform decisions about enhanced surveillance.
- The National Health Service has remained under strain after March 2023, further evaluation of surgical pathway completion in infants with CHD is needed.

## Introduction

Congenital heart disease (CHD) affects approximately 5600 live-born children annually in England and Wales.^1^ Each year, 7000–8000 paediatric cardiac procedures are undertaken in the UK, 58%–60% of them in children under 1 year old (infants),^2^ with an average 30-day mortality rate of 2%,^2^ although the risk is higher for complex CHD.^3^ The risk of mortality for an individual child is greatest during infancy.^1^ There is a substantial risk of postdischarge mortality and unexpected critical illness, especially in medically complex infants.^4^ Studies from the USA indicate that postdischarge mortalities can be mitigated by increased healthcare surveillance.^5^ In the USA, risk factors for late death in infants with complex CHD include residence in more deprived neighbourhoods,^6^ Hispanic compared with white ethnicity^7^ and black compared with white ethnicity.^8^ Poorer outcomes based on social factors have been attributed to unequal access to healthcare.^6 8^

Although the UK National Health Service (NHS) provides care that is universal and free at the point of access, services were affected by the COVID-19 pandemic. During pandemic restrictions, some UK-based families and patients affected by CHD reported delays and cancellations in healthcare appointments in an online forum study.^9^ The number of elective paediatric cardiac surgeries undertaken was reduced, during pandemic restrictions, although urgent surgeries were maintained.^10^ The parents of young children were in general, much less likely than usual to access emergency care.^11^ We, therefore, aimed to explore the impact of the COVID-19 pandemic on the timing of the expected operative treatment pathway and to evaluate any increases in mortality or time spent in hospital during infancy for children with complex CHD. In secondary analyses, we aimed to investigate whether social covariates that have been linked vulnerability of infants with CHD (sex,^12^ ethnicity^7 8^ and residential area deprivation^6^) were associated with the study outcomes during the pandemic.

## Method

### Study design

We conducted an observational cohort study based on prospectively recorded electronic health record data: (1) National Congenital Heart Diseases Audit (NCHDA) (the core dataset), (2) General Practice Extraction Service Data for Pandemic Planning and Research (GDPPR), (3) Hospital Episode Statistics (HES) and (4) Office of National Statistics (ONS) mortality data. The deidentified data were securely accessed through NHS England’s Secure Data Environment service for England via the BHF Data Science Centre’s CVD-COVID-UK/COVID-IMPACT Consortium (https://bhfdatasciencecentre.org/areas/cvd-covid-uk-covid-impact/).^13^ NHS England implemented strict disclosure control measures to safeguard against the release of personal, sensitive and confidential information.^14^ This includes providing only the month and year of birth, suppressing counts if fewer than 10 patients, and rounding counts to the nearest multiple of 5 otherwise.

### Patient and public involvement

We worked with CHD user groups (Little Hearts Matter, the Children’s Heart Federation and for adults with CHD, the Somerville Foundation), and with patient coresearchers affected by CHD, to select the sentinel CHDs used in this study.^15^ Parents and users told our study team that delays in treatment, mortality and prolonged hospital stay are important outcomes. Our study was reviewed by the patient and user panel of the BHF Data Science Centre’s CVD-COVID-UK/COVID-IMPACT consortium.

### Data management

**Figure 1:**
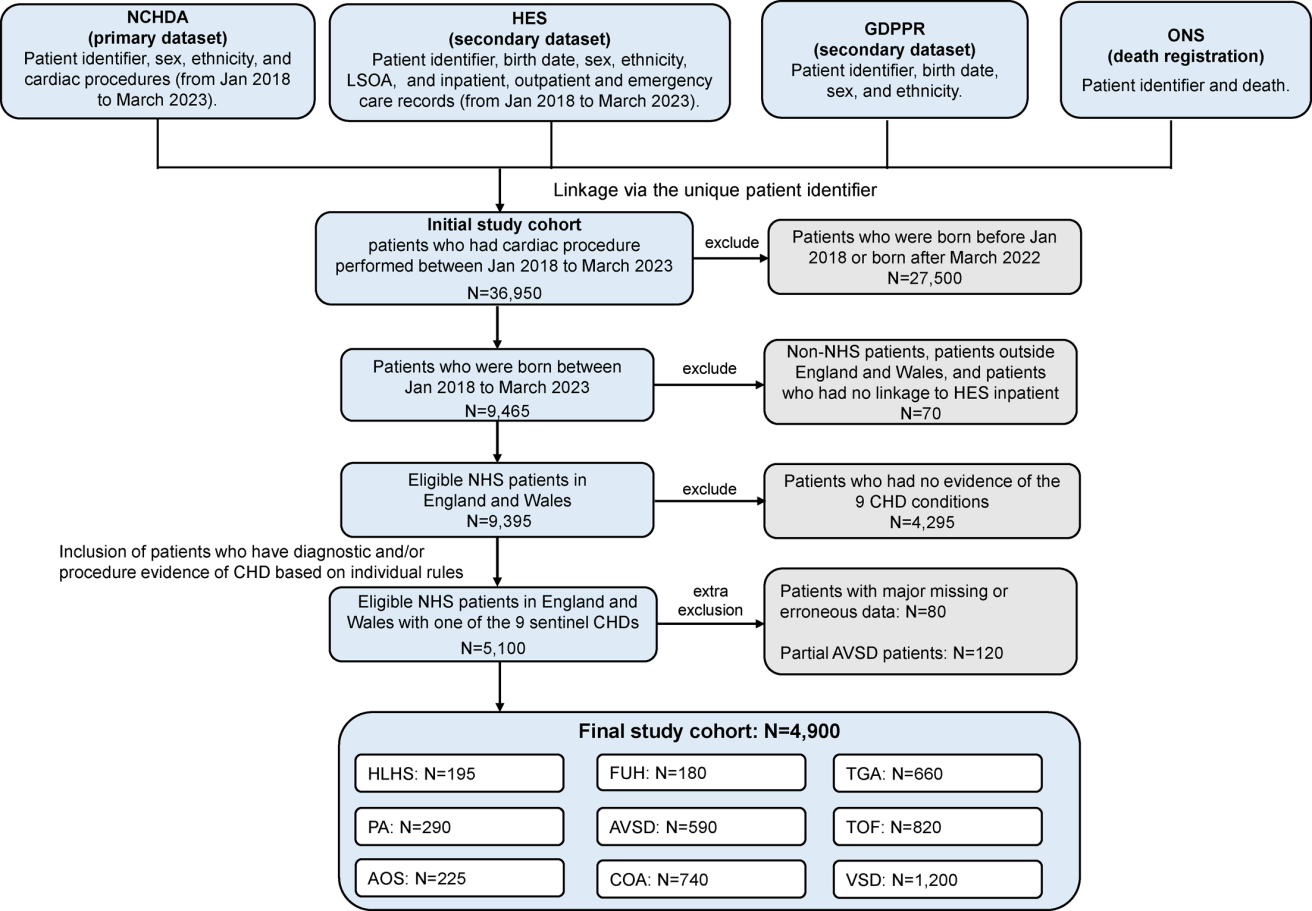
Inclusion and exclusion flow chart. All data were retrieved in July 2023 through the Secure Data Environment service for England within the National Health Service (NHS) of England. AOS, congenital aortic stenosis; AVSD, atrioventricular septal defect; COA, coarctation of the aorta; FUH, functionally univentricular heart; GDPPR, General Practice Extraction Service Data for Pandemic Planning and Research; HES, Hospital Episode Statistics; HLHS, hypoplastic left heart syndrome; LSOA, lower layer super output areas; NCHDA, National Congenital Heart Diseases Audit; ONS, Office of National Statistics; PA, pulmonary atresia; TGA, transposition of the great arteries; TOF, tetralogy of Fallot; VSD, ventricular septal defect.

We created a patient-level dataset (figure 1) using records of cardiac surgical and interventional catheterisation procedures from NCHDA linked using the unique patient identifier to death registrations from ONS; primary care records in GDPPR and HES routine administrative data. All clinical data were organised into ‘care spells’ that may include procedures, inpatient stays, outpatient visits or accident and emergency (A&E) visits in any combination to manage overlaps in time frames.^16^

### Sentinel CHDs

In a prior research study, we selected and characterised ‘sentinel CHDs’ which are a consistently defined group of major CHDs suited for long-term monitoring using NCHDA.^15^ Sentinel CHDs were selected considering clinician, patient and analytical perspectives, based on their prevalence and significant impacts on infants of early interventions and mortalities. Ordered by complexity these are hypoplastic left heart syndrome (HLHS), functionally univentricular heart conditions (FUH), of double inlet left ventricle and tricuspid atresia, pulmonary atresia all types (PA), transposition of the great arteries (TGA), tetralogy of Fallot (TOF), atrioventricular septal defect (AVSD, including complete AVSD, unbalanced AVSD and tetralogy AVSD, but excluding partial AVSD), congenital aortic stenosis (AOS), coarctation of the aorta (COA) and significant ventricular septal defect (VSD). Each of these CHDs has defined subgroups as defined previously^15^ displayed in online supplemental table S1.

### Inclusion and exclusion criteria

We included patients with a sentinel CHD who were born between January 2018 (to ensure complete procedure history) and March 2022 (to ensure at least 1 year of follow-up) and had a cardiac procedure. We excluded patients who had no linkage to ONS death registration or HES data (those from overseas, Scotland and Northern Ireland).

### Expected operative treatment

For each sentinel CHD, we identified the expected interventional treatment pathways in terms of cardiac surgery, interventional catheterisation procedures and hybrid types based on previously defined algorithms using diagnosis and procedure codes.^15^ For functionally single ventricle (f-SV) CHDs, the expected treatment pathway consists of a series of exclusively palliative procedures,^15^ hence we identified the expected pathway in infancy as ‘palliative stage 1 procedures’ and ‘stage 2 Glenn procedures’. For biventricular CHDs, the expected pathway involves a ‘reparative surgery’, and potentially also a ‘palliative stage 1 procedure’,^15^ hence these were identified. We did not consider prepathway procedures and reinterventions (as previously defined^15^) in this study.

### Exposure of interest: birth era

We defined birth eras informed by key dates related to the pandemic^10^

- Prepandemic (reference): Patients born from January 2018 to March 2019, with care in infancy unaffected by the pandemic.
- Transition period: Patients born from April 2019 to March 2020, who may have been affected during infancy by the start of the pandemic.
- Restrictions: Patients born from April 2020 to June 2021; we collapsed the three restriction and corresponding relaxation periods due to limited sample size.
- Postrestrictions: Patients born from July 2021 when restrictions were eased in England and Wales, until March 2022, the latest feasible limit of the data sources.

### Study outcomes

1. Observed ages at treatment pathway operations: There are no accepted ‘gold standard’ ages for treatment pathway procedures therefore, we used prepandemic procedure ages as the ‘proxy’ gold standard since this reflects an era when the service was running normally. Of note, birth dates were provided as month and year only.
2. Mortality rate at the age of 1 year (infant mortality).
3. Hospital care utilisation in infancy: We categorised hospital utilisation into three types for descriptive purposes: total (inpatient, outpatient and A&E), inpatient and outpatient. Our focus for hypothesis testing was inpatient days.

### Participant characteristics/risk factors

We extracted a series of variables to describe patient characteristics and risk factors.^17^ Casemix was defined based on the specific CHD subtype,^15^ the presence of extracardiac anomalies (eg, genetic syndrome) and prematurity (birth at gestation less than 37 weeks). We defined social factors of sex, socioeconomic status and ethnic group. To describe socioeconomic status, we used the Index of Multiple Deprivation (IMD) 2019 derived from HES, coded as lower layer super output area level, dividing this into equal quintiles (IMD 1–5). For ethnicity, we prioritised data from GDPPR and classified this as white, Asian, black, mixed and other. If a GDPPR ethnicity record was not available, we used HES and then NCHDA to assign ethnic group. In the analyses with low number of events, we collapsed CHD type, ethnicity and socioeconomic status into larger categories: sentinel CHD type without subgroups, white versus non-white (black, Asian, mixed and other) and deprived areas (IMD 1–2) vs non-deprived areas (IMD 3–5).

### Statistical analyses

#### Question 1: were outcomes poorer for infants born during the pandemic compared with those born prepandemic?

Descriptive statistics were presented as numbers and percentages, or as median and IQRs. We reported the study outcomes by each birth era and by sentinel CHDs.

To evaluate the outcome of age at treatment pathway procedures, which are strongly linked to CHD type and urgency,^2^ we grouped operations as (1) palliative stage 1 procedures, which are urgent procedures for critical CHD and (2) f-SV stage 2 procedures and reparative procedures, many of which involve admission from home. To best approximate patients’ age at treatment based on the month and year of birth provided, we set birthdays to the 1st or the 15th of the month, depending on whether the patients’ first hospital admission was in the first or second fortnight of the birth month.

We evaluated the outcome of infant mortality (ie, cumulative mortality rate at 1 year old) using the Kaplan-Meier estimator.

We evaluated the outcome of hospital care utilisation (inpatient, outpatient) and days spent at home in infancy (ie, 365 days—total days spent as an inpatient; patients who died before age 1 year were assigned as 0 days at home as the worst outcome).

There was a small amount of missing data for ethnicity (65 (1.3%)) and area deprivation (25 (0.6%)). We only included those with complete data in the analyses, for example, 4815 (98.3%) of the total cohort.

We used the Wilcoxon rank sum test to assess delays in procedure timing and differences in hospital stay lengths between each pandemic period and the prepandemic baseline.

We explored the relationship between the exposure variable of birth era and outcomes using univariable and multivariable models (quantile regression for the median age at the two types of pathway procedures and median days at home by age 1 year, logistic regression for infant mortality), and including other risk factors (casemix and social factors) in the multivariable models.

#### Question 2: were any study outcomes poorer based on social factors either overall or by birth era?

First, we explored the associations of social factors with the study outcomes using univariable and multivariable models (using the same risk factors as in question 1). Then we explored the associations of sex, residential area deprivation and ethnicity with each outcome by fitting interaction terms with the birth era exposure in the multivariate models, to assess whether those children with recognised vulnerabilities^46^,^8 12 18^ (girls, high deprivation and ethnic minority background) were affected by changes to services in the pandemic more than children without these attributes. A likelihood ratio test of nested models was used to determine statistical evidence of incorporating these interactions, and a p value less than 0.05 was considered as statistical significance.

This analysis followed a preset plan published on GitHub, including the rules for the assignment of CHDs and the analysis code (https://github.com/BHFDSC/CCU007_03).

Data management and statistical analyses was performed with Stata V.15 software (StataCorp) and R (V.4.3.0, Foundation for Statistical Computing, Vienna, Austria).

## Results

### The study population

The cohort consisted of 4900 children, of whom 1545 (31.5%) patients were born prepandemic, 1175 (24.0%) were born in a transition period, 1375 (28.0%) were born during pandemic restrictions and 810 (16.5%) were born postrestrictions . The casemix was HLHS (195; 3.9%), FUH (180; 3.7%), TGA (610, 13.5%), PA (290; 5.9%), AVSD (590; 12.1%), TOF (820; 16.7%), AOS (225; 4.6%), COA (740; 15.1%) and VSD (1200; 24.5%). The sentinel CHD subgroups are presented by birth era in online supplemental table S2; 695 children (14.1%) were born preterm, and 1430 (29.2%) had congenital comorbidities including 675 with Down syndrome. The social factors indicated that most children were white (3570; 72.8%) or Asian (mainly south Asian) (665; 13.6%); more than half, 2545 (51.9%), lived in deprived areas (IMD 1–2) and 2810 (57.4%) were boys (table 1).

**Table 1:** Characteristics of the study cohort (n=4900)

Non-clinical	Birth era	N (%)
	Prepandemic baseline (January 2018–March 2019)	1545 (31.5)
	Transition period: (April 2019–March 2020)	1175 (24.0)
	Resctriction period: (April 2020–June 2021)	1375 (28.0)
	Postrestriction period: (July 2021–March 2022)	810 (16.5)
	**Gender**	
	Male	2810 (57.4)
	Female	2090 (43.6)
	**Ethnic group**	
	White	3570 (72.8)
	Non-white	
	Black (African/Caribbean)	220 (4.5)
	Asian	665 (13.6)
	Mixed/other	380 (7.8)
	Missing	65 (1.3)
	**IMD (area deprivation) score**	
	Deprived area (quintile 1-2)	
	Quintile 1 (most deprived)	1435 (29.3)
	Quintile 2	1110 (22.6)
	Non-deprived area (quintile 3-5)	
	Quintile 3	925 (18.8)
	Quintile 4	750 (15.3)
	Quintile 5 (least deprived)	655 (13.3)
	Missing	25 (0.6)
**Clinical**	**CHD diagnosis (in order of decreasing complexity)**	
	Hypoplastic left heart syndrome	195 (3.9)
	Functionally univentricular heart	180 (3.7)
	Transposition of the great arteries	660 (13.5)
	Pulmonary atresia	290 (5.9)
	Atrioventricular septal defect (AVSD)	590 (12.1)
	Tetralogy of Fallot	820 (16.7)
	Congenital aortic stenosis	225 (4.6)
	Coarctation of the aorta	740 (15.1)
	Significant ventricular septal defect (VSD)	1200 (24.5)
	**Preterm birth (before37 weeks)**	695 (14.1)
	**Congenital noncardiac comorbidity** *	1430 (29.2)

Characteristics of the study cohort (n=4900).

* Including 675 Down syndrome and most of them were atrioventricular septal defect (AVSD, n=450) or ventricular septal defect (VSD, n=205).

CHDcongenital heart diseaseIMDIndex of Multiple Deprivation

### Question 1: study outcomes by birth era

#### Age at treatment pathway procedures

**Figure 2:**
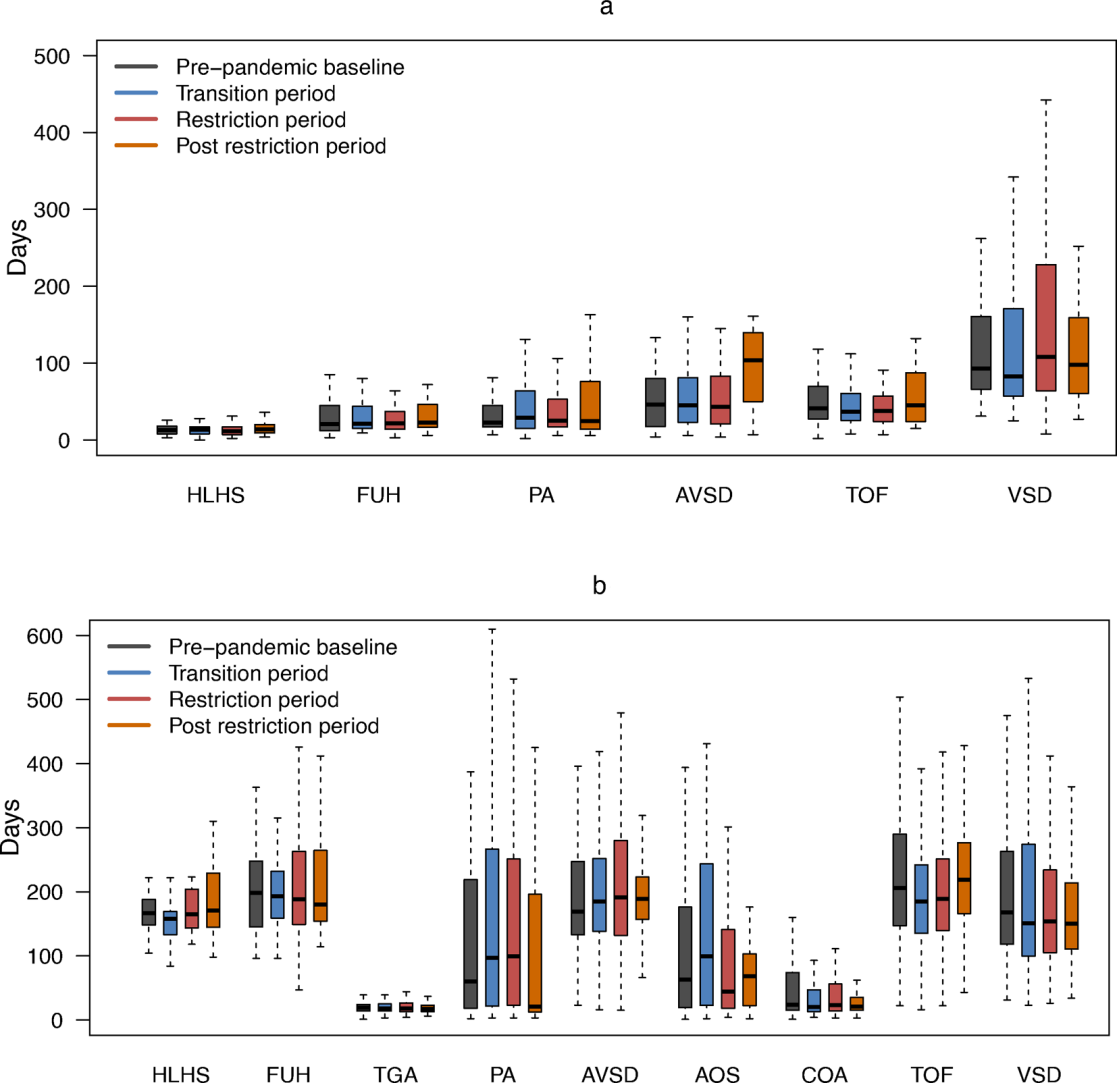
Boxplots depicting age of pathway procedures since birth, by birth era. (**a**) Boxplots of age at palliative stage 1 procedure (age at palliative stage 1 in TGA, AOS and COA was not shown due to limited sample size when broken down by era (n<10)); (**b**) boxplots of age at palliative stage 2 or reparative procedure. There were 15 patients who had both a reparative procedure and a single ventricle stage 2 (CHD subgroups: PA, AVSD and TOF), and their first occurring procedures were included in **b**. Boxplots show the median (horizontal line inside the box), the interquartile range (box) and whiskers that extend from the box to the minimum and maximum values within 1.5 times the IQR from the first quartile and third quartile, respectively. Detailed data are presented in online supplemental table S3, and test results of statistical evidence for a delay of procedure timing between each pandemic era compared with the prepandemic baseline are presented in online supplemental table S4. AOS, congenital aortic stenosis; AVSD, atrioventricular septal defect; COA, coarctation of the aorta; FUH, functionally univentricular heart; HLHS, hypoplastic left heart syndrome; PA, pulmonary atresia; PS, pulmonary stenosis; TGA, transposition of the great arteries; TOF, tetralogy of Fallot; VSD, ventricular septal defect.

**Figure 3:**
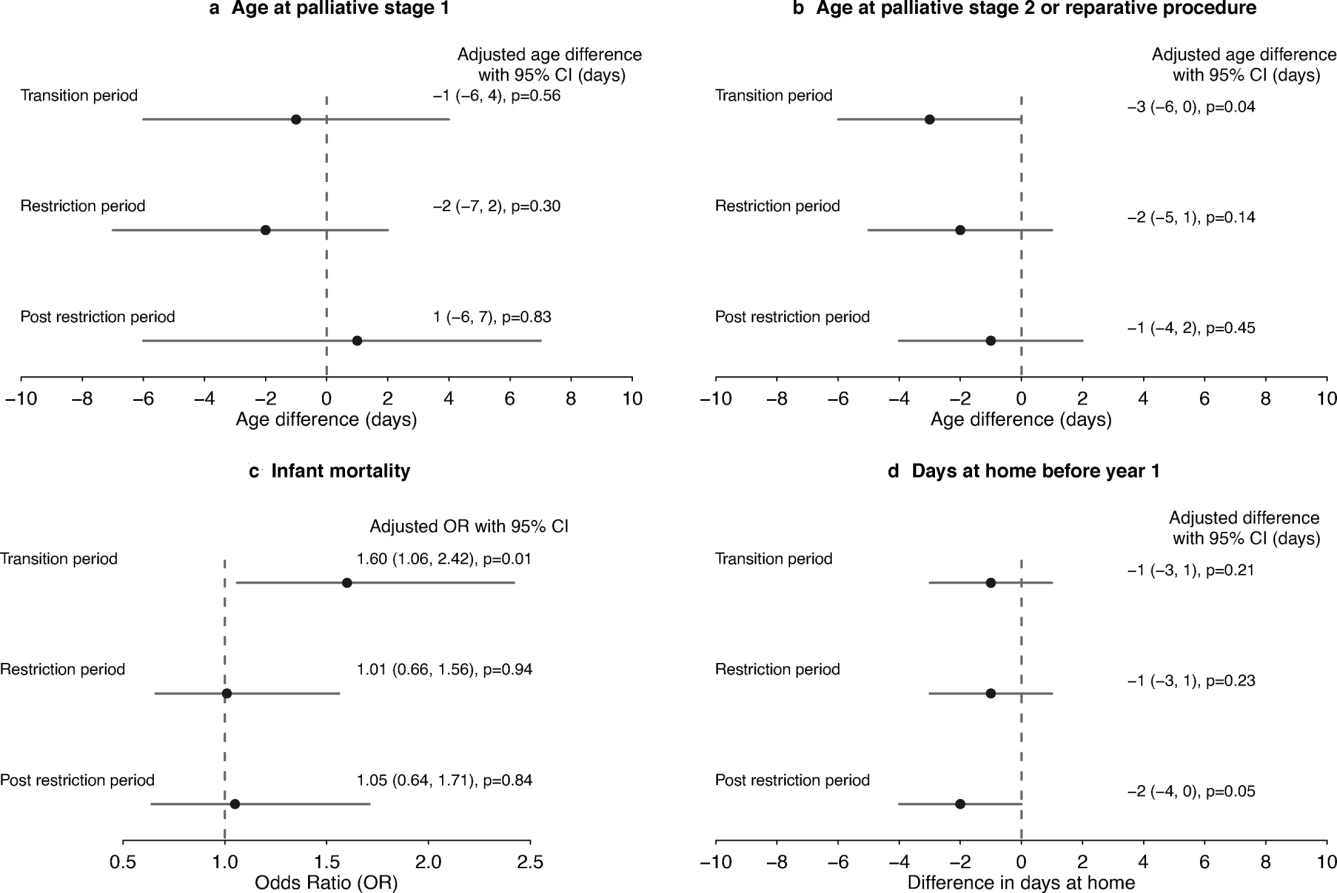
Forest plot for all modelling outcomes related to birth era. (**a**) Adjusted median age at palliative stage 1 procedure; (**b**) adjusted median age at palliative stage 2 or reparative procedure; (**c**) adjusted OR for infant mortality (death under age 1 year old) and (**d**) adjusted median days spent at home before age 1 year old. Reference group was prepandemic baseline in all models. Complete data analysis was performed. Univariate results and results for other adjusted covariates are presented in online supplemental table S5, S7 and S12.

4830 (98.4%) children underwent at least one treatment pathway procedure (the remainder had a prepathway procedure only, eg, balloon atrial septostomy). Age at pathway procedures by birth era for each sentinel CHD varied widely by CHD subtype (figure 2, online supplemental table S3). There was no evidence of delay in the ages at which treatment procedures were undertaken during the pandemic eras compared with the reference prepandemic period (Wilcoxon rank sum test p>0.39 (online supplemental table S4). After adjustment for age, sex, casemix and social factors, we observed no delay in median pathway procedure ages during the pandemic eras compared with prepandemic, although patients born in the transition era had palliative stage 2/reparative procedures at a slightly younger age (adjusted difference in days −3 (95% CI −6, 0), p=0.04 (figure 3a,b, online supplemental table S5).

#### Infant mortality

**Figure 4:**
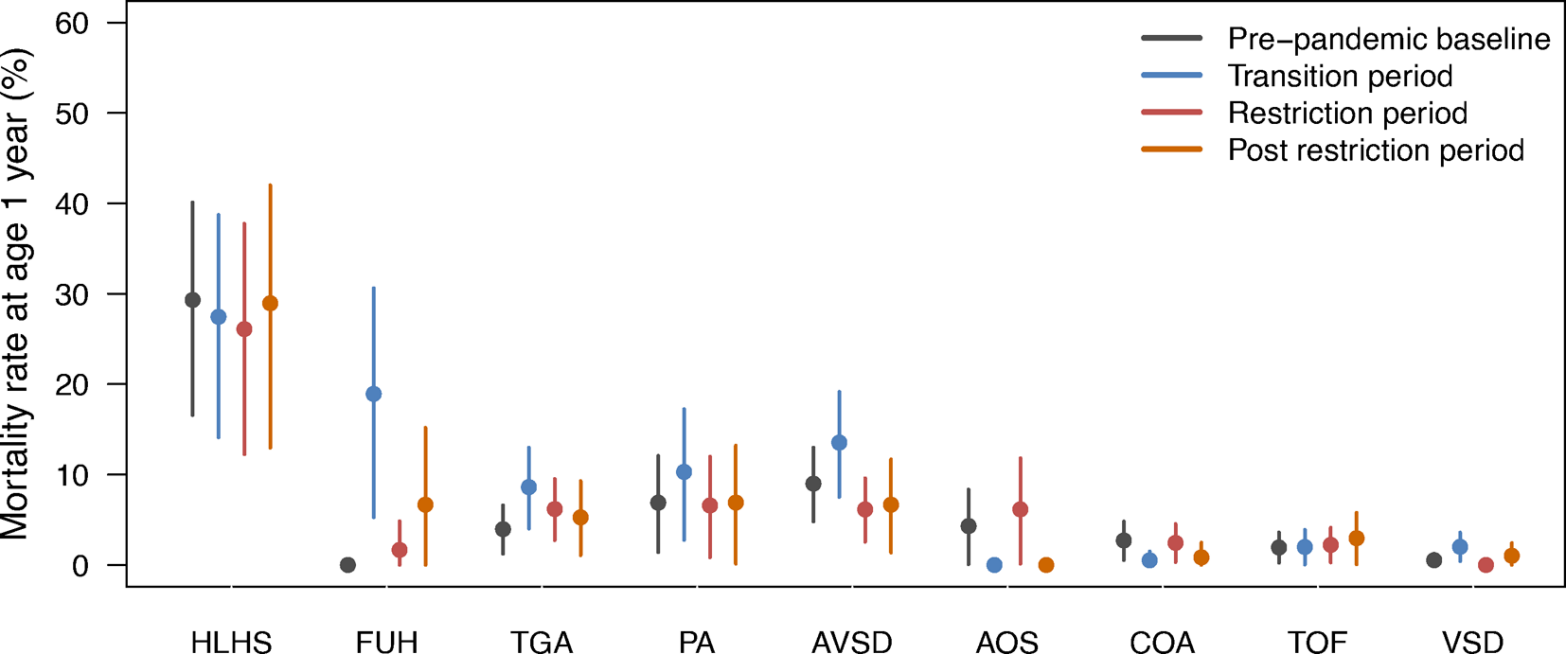
Mortality rate at 1 year (using Kaplan-Meier) with 95% CI by birth era and CHD diagnoses. Detailed data are presented in online supplemental table S6. AOS, congenital aortic stenosis; AVSD, atrioventricular septal defect; CHD, congenital heart disease; COA, coarctation of the aorta; FUH, functionally univentricular heart; HLHS, hypoplastic left heart syndrome; PA, pulmonary atresia; TGA, transposition of the great arteries; TOF, tetralogy of Fallot; VSD, ventricular septal defect.

Infant mortality varied widely by individual CHD subtype: in the most complex CHD, HLHS, it was 28.0% (21.4%, 34.0%) and in the least complex CHD, VSD, it was 0.8% (95% CI 0.3%, 1.3%) (figure 4). There was no evidence of increasing rates of infant mortality for those born during the pandemic eras: 1-year mortality rate prepandemic 4.2% (95% CI 3.2%, 5.2%), transition 6.0% (4.6%, 7.3%), restrictions 4.0% (3.0%, 5.0%) and postrestrictions 4.5% (3.0%, 5.9%) (figure 4, online supplemental table S6). After adjusting for casemix and social factors, we observed modestly higher rates of infant mortality among those born during the transition era (adjusted OR 1.60 (95% CI 1.06, 2.42), p=0.01) (figure 3c and online supplemental table S7).

#### Hospital care utilisation

**Figure 5:**
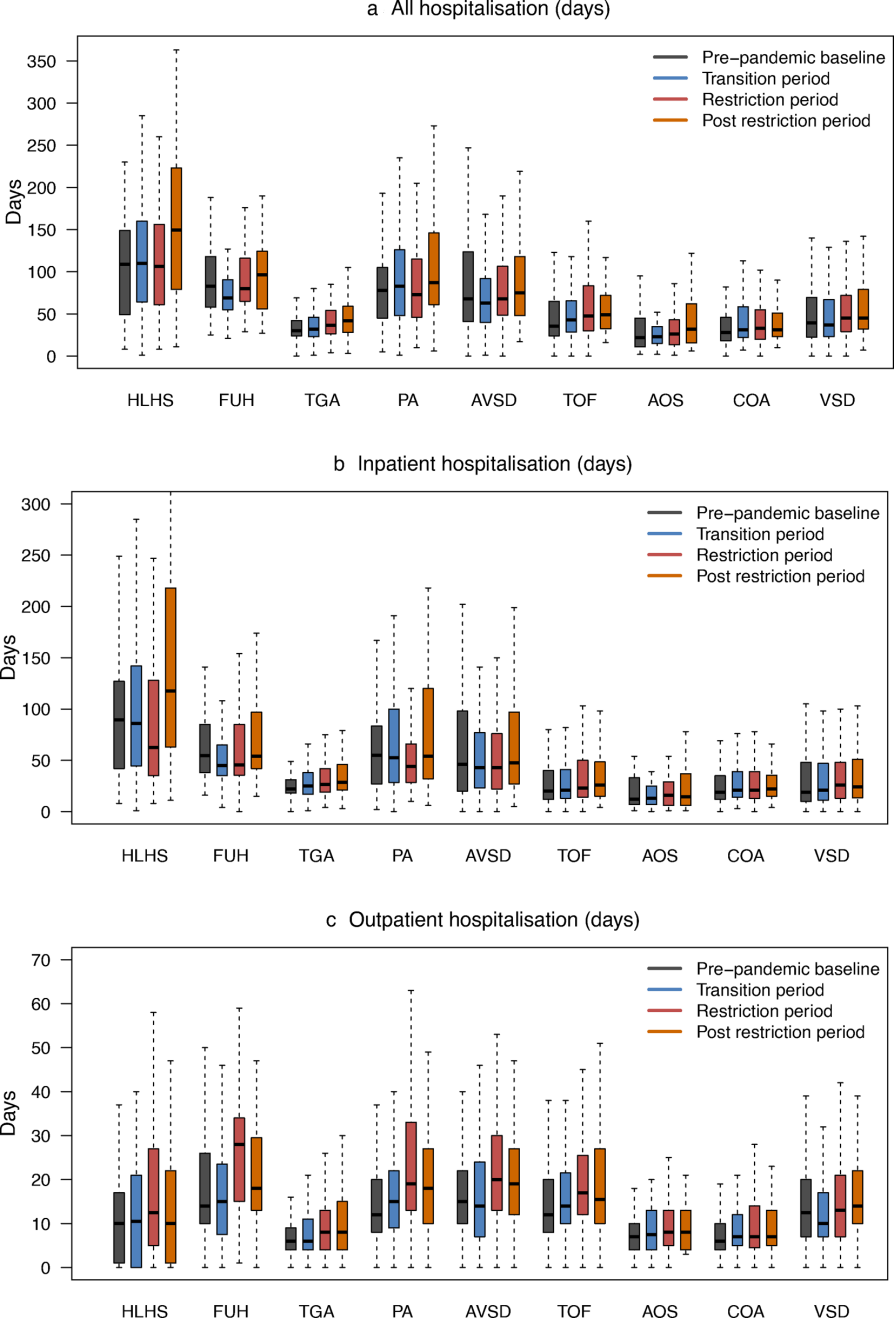
Boxplots depicting length of hospital stay before the age of 1 year by birth era and CHD diagnoses. (**a**) Total days spent in the hospital (inpatient, outpatient and accident and emergency visits); (**b**) Number of inpatient days; (**c**) Number of outpatient days. All panels show the median (horizontal black line line), IQR (coloured solid bars bars) and 1.5×IQR (dotted vertical lines). Outliers outside these limits are not shown. Corresponding numbers are detailed in online supplemental table S6–S8. Test results of statistical evidence for any difference of hospital stay between each pandemic era compared with the prepandemic baseline are presented in online supplemental table S8. Detailed data are presented in online supplemental table S9–S11. AOS, congenital aortic stenosis; AVSD, atrioventricular septal defect; CHD, congenital heart disease; COA, coarctation of the aorta; FUH, functionally univentricular heart; HLHS, hypoplastic left heart syndrome; PA, pulmonary atresia; TGA, transposition of the great arteries; TOF, tetralogy of Fallot; VSD, ventricular septal defect.

We observed changes in hospital care utilisation over time (figure 5), with total hospital contact days ranging from 40 (IQR: 24–76) and 41 (25–71) days in prepandemic and transition eras, increasing to 47 (29–80) and 50 (31–92) in restrictions and postrestrictions eras (Wilcoxon rank sum test p<0.001 for restrictions and postrestrictions compared with baseline online supplemental table S8. We observed similar findings for inpatient stays and outpatient consultations (online supplemental tables S9–S11).

#### Days spent at home in infancy

After accounting for casemix and social factors, there was no evidence of fewer days at home for children born in the transition or restriction eras (p=0.22 and p=0.21, figure 3d, but children born postrestrictions had fewer days at home in infancy (adjusted difference −2 days (95% CI −4, 0), p=0.05), compared with children born prepandemic (online supplemental table S12).

### Question 2: study outcomes and social factors

Associations between birth era and age at pathway procedure, infant mortality or days at home in infancy did not differ significantly by subgroups of ethnicity, deprivation or sex (likelihood test for nested model p>0.05 when incorporating these interactions).

Social factors with all birth eras combined showed the following notable results with respect to sex, ethnic group and neighbourhood deprivation (see online supplemental tables S5, S7 and S12). Detailed descriptive data are provided in online supplemental tables S13–S15.

Patient sex was unrelated to any of the three outcomes, adjusted for casemix.

The results raised the possibility of ethnic disparities: age at palliative stage 1 procedure was older among Asian children than white children, adjusted difference in days 6 (95% CI 0, 12), p=0.05). Children of black ethnicity (adjusted difference −7 (−12, –1), p=0.01) and children of Asian ethnicity (adjusted difference −3 (−6, 0), p=0.02) spent fewer median days at home in infancy than children with white ethnicity. However, there were no differences between the non-white and the white ethnic groups for infant mortality (p=0.26).

There was evidence for socioeconomic disparity as children in the most deprived binary category had considerably higher rates of infant mortality compared with those in the least-deprived (adjusted OR 1.56 (95% CI 1.11, 2.18), p=0.004). There was also a gradient in the number of days spent at home across the quintiles of deprivation, with children resident in the most deprived neighbourhood quintile spending the fewest days at home (eg, adjusted difference in days −4 (95% CI −6, –2), p<0.001) (reference least deprived quintile).

## Discussion

### Summary and interpretation

Our study, which aimed to explore any health service impacts from the COVID-19 pandemic on the treatment pathways and outcomes of infants with complex CHD encouragingly, found no evidence that infants with sentinel CHDs experienced delays to their pathway interventions during the pandemic nor immediately after the pandemic, when the healthcare system remained under strain. There was evidence that children born in the transitional era had a slightly higher mortality than children born in other eras, yet most of these deaths occurred before the pandemic started and the clinical significance is unclear. There was no evidence that the pandemic restrictions were linked to increases in mortality for infants with CHD, implying that their safety was preserved.

Our study shows clear changes in hospital care utilisation related to the pandemic among infants with CHD: increased outpatient contacts, often as remote appointments, were used as check-ups, to monitor these fragile children. Hospital inpatient stays increased from baseline among those born during restrictions and then were at their highest among those born after pandemic restrictions ended. The increased inpatient stays could be an indication of poorer health due to viral infections given that studies indicate young children may have experienced respiratory viral infections more severely postpandemic.^19 20^ Infants with CHD are particularly vulnerable to respiratory viruses^21 22^ and are more likely to be hospitalised with SARS-CoV-2 than older children.^23^

We observed significant socioeconomic disparities in hospital care utilisation and rates of infant mortality. Ethnic disparities were also apparent, although largely restricted to hospital care utilisation. Socioeconomic and ethnic disparities did not appear to have changed during the pandemic. These findings contrast with earlier studies of complex CHD from England, when no such differences were detected,^24^,^26^ and could indicate that disparities have widened over time. Health inequalities for children with CHD are observed in the USA, where minority race and neighbourhood deprivation have been repeatedly linked to poorer outcomes in children with CHD.^826^,^29^

### Strengths and limitations

A strength of our study was its inclusive use of population-based linked health record data. Nonetheless as NCHDA is a procedure-based registry, we only considered children who underwent at least one intervention for CHD. Since the pandemic was a recent event, our study was only able to consider outcome at 1 year of age for children with sentinel CHDs. Because more urgent procedures (including many infant operations)^10^ were prioritised during pandemic restrictions, it is possible that older children experienced delays and changes in care that our study was not able to investigate.

## Conclusions

The first year of life is a period of vulnerability for children with CHD, who require key treatment pathway procedures and regular healthcare maintenance. Specialist services for CHD performed well during the pandemic, in the sense that there were no delays in time-critical surgical pathway procedures for infants and infant mortality rate remained low. Further research is needed to elucidate the reasons underlying the observed increase in hospital care utilisation among infants with CHD, especially postrestrictions, and to better understand and address socioeconomic and ethnic disparities in healthcare utilisation and infant mortality.

## supplementary material

10.1136/openhrt-2024-002964 Supplementary file 1

## Data Availability

Data may be obtained from a third party and are not publicly available.

## References

[1] Billett J, Majeed A, Gatzoulis M (2008). Trends in hospital admissions, in-hospital case fatality and population mortality from congenital heart disease in England, 1994 to 2004. Heart.

[2] National Congenital Heart Diseases Audit N (2023). National cardiac audit programme.

[3] Crowe S, Brown KL, Pagel C (2013). Development of a diagnosis- and procedure-based risk model for 30-day outcome after pediatric cardiac surgery. J Thorac Cardiovasc Surg.

[4] Crowe S, Ridout DA, Knowles R (2016). Death and Emergency Readmission of Infants Discharged After Interventions for Congenital Heart Disease: A National Study of 7643 Infants to Inform Service Improvement. J Am Heart Assoc.

[5] Rudd NA, Frommelt MA, Tweddell JS (2014). Improving interstage survival after Norwood operation: outcomes from 10 years of home monitoring. J Thorac Cardiovasc Surg.

[6] Sengupta A, Bucholz EM, Gauvreau K (2023). Impact of Neighborhood Socioeconomic Status on Outcomes Following First-Stage Palliation of Single Ventricle Heart Disease. J Am Heart Assoc.

[7] Ghanayem NS, Allen KR, Tabbutt S (2012). Interstage mortality after the Norwood procedure: Results of the multicenter Single Ventricle Reconstruction trial. J Thorac Cardiovasc Surg.

[8] Lopez KN, Morris SA, Sexson Tejtel SK (2020). US Mortality Attributable to Congenital Heart Disease Across the Lifespan From 1999 Through 2017 Exposes Persistent Racial/Ethnic Disparities. Circulation.

[9] Wray J, Pagel C, Chester AH (2021). What was the impact of the first wave of COVID-19 on the delivery of care to children and adults with congenital heart disease? A qualitative study using online forums. BMJ Open.

[10] Karthikeyan Suseeladevi A, Denholm R, Babu-Narayan S (2024). Impact of covid-19 pandemic on rates of congenital heart disease procedures among children: prospective cohort analyses of 26,270 procedures in 17,860 children using cvd-covid-uk consortium record linkage data. Cardiovascular Med.

[11] Honeyford K, Coughlan C, Nijman RG (2021). Changes in Emergency Department Activity and the First COVID-19 Lockdown: A Cross-sectional Study. West J Emerg Med.

[12] Almossawi O, O’Brien S, Parslow R (2021). A study of sex difference in infant mortality in UK pediatric intensive care admissions over an 11-year period. Sci Rep.

[13] Trusted research environment service for England. https://digital.nhs.uk/coronavirus/coronavirus-data-services-updates/trusted-research-environment-service-for-england.

[14] Wood A, Denholm R, Hollings S (2021). Linked electronic health records for research on a nationwide cohort of more than 54 million people in England: data resource. BMJ.

[15] Brown KL, Huang Q, Espuny-Pujol F (2024). Evaluating Long-Term Outcomes of Children Undergoing Surgical Treatment for Congenital Heart Disease for National Audit in England and Wales. J Am Heart Assoc.

[16] Espuny Pujol F, Pagel C, Brown KL (2022). Linkage of National Congenital Heart Disease Audit data to hospital, critical care and mortality national data sets to enable research focused on quality improvement. BMJ Open.

[17] Brown KL, Rogers L, Barron DJ (2017). Incorporating Comorbidity Within Risk Adjustment for UK Pediatric Cardiac Surgery. Ann Thorac Surg.

[18] Gilboa SM, Salemi JL, Nembhard WN (2010). Mortality resulting from congenital heart disease among children and adults in the United States, 1999 to 2006. Circulation.

[19] Kurz H, Sever-Yildiz G, Kocsisek CV (2024). Respiratory Syncytial Virus and Influenza During the COVID-19 Pandemic: A Two-center Experience. Pediatr Infect Dis J.

[20] Cai W, Köndgen S, Tolksdorf K (2024). Atypical age distribution and high disease severity in children with RSV infections during two irregular epidemic seasons throughout the COVID-19 pandemic, Germany, 2021 to 2023. Euro Surveill.

[21] Abud KCO, Machado CM, Vilas Boas LS (2023). Respiratory viruses and postoperative hemodynamics in patients with unrestrictive congenital cardiac communications: a prospective cohort study. Eur J Med Res.

[22] Silva TH da, Pinho JRR, da Silva Junior TJ (2019). Epidemiology of viral respiratory infections in children undergoing heart surgery. Prog Pediatr Cardiol.

[23] Wilde H, Tomlinson C, Mateen BA (2023). Hospital admissions linked to SARS-CoV-2 infection in children and adolescents: cohort study of 3.2 million first ascertained infections in England. BMJ.

[24] Rogers L, Pagel C, Sullivan ID (2018). Interventional treatments and risk factors in patients born with hypoplastic left heart syndrome in England and Wales from 2000 to 2015. Heart.

[25] Hadjicosta E, Franklin R, Seale A (2022). Cohort study of intervened functionally univentricular heart in England and Wales (2000-2018). Heart.

[26] Knowles RL, Ridout D, Crowe S (2019). Ethnic-specific mortality of infants undergoing congenital heart surgery in England and Wales. Arch Dis Child.

[27] Davey B, Sinha R, Lee JH (2021). Social determinants of health and outcomes for children and adults with congenital heart disease: a systematic review. Pediatr Res.

[28] de Loizaga SR, Schneider K, Beck AF (2022). Socioeconomic Impact on Outcomes During the First Year of Life of Patients with Single Ventricle Heart Disease: An Analysis of the National Pediatric Cardiology Quality Improvement Collaborative Registry. Pediatr Cardiol.

[29] Ludomirsky AB, Bucholz EM, Newburger JW (2021). Association of Financial Hardship Because of Medical Bills With Adverse Outcomes Among Families of Children With Congenital Heart Disease. JAMA Cardiol.

