# Supplementary material for: Peripandemic outcomes of infants treated for sentinel congenital heart diseases in England and Wales
Source: Open Heart. 2025 Feb 17;12(1):e002964. doi: 10.1136/openhrt-2024-002964 (PMC11836792; doi:10.1136/openhrt-2024-002964)
Supplement: Supplementary file 1 [file openhrt-12-1-s001.docx]

# Supplemental materials for the manuscript: Peri-pandemic outcomes of infants treated for sentinel congenital heart diseases in England and Wales

## Supplementary Table S1: Sentinel CHD diagnoses with subgroups.

*we excluded partial AVSD because age at repair is well over 1 year old.

| **CHD diagnosis (in decreasing order of clinical complexity)** | **CHD subgroup** |
| --- | --- |
| Functional single ventricle conditions | |
| Hypoplastic left heart syndrome (HLHS) | HLHS |
| Functionally univentricular heart (FUH) | Double inlet ventricle |
|  | Tricuspid atresia |
| Conditions where a range of anatomy occurs (either a single ventricle or a reparative pathway can occur) | |
| Transposition of the great arteries (TGA) | Complex TGA with PS |
|  | Complex TGA without PS |
|  | TGA with intact ventricular septum |
| Pulmonary atresia (PA) | PA with VSD |
|  | PA with intact ventricular septum |
| Atrioventricular septal defect (AVSD*) | Tetralogy with AVSD |
|  | Unbalanced AVSD |
|  | Complete AVSD |
| Biventricular conditions | |
| Tetralogy of Fallot (TOF) | Tetralogy absent pulmonary valve |
|  | Tetralogy with DORV |
|  | Standard tetralogy |
| Aortic stenosis (AOS) | AOS with multi-level left heart obstruction |
|  | Isolated AOS |
| Coarctation of the aorta (COA) | Coarctation with VSD |
|  | Isolated COA |
| Ventricular septal defect (VSD) | Multiple VSDs |
|  | Single VSD |

## Supplementary Table S2: Breakdown of patients’ number by diagnosis subtype and birth era.

AOS=congenital aortic stenosis; AVSD=atrioventricular septal defect; COA= coarctation of the aorta; DORV=double outlet right ventricle; FUH=functionally univentricular heart; HLHS=hypoplastic left heart syndrome; TGA=transposition of the great arteries; TOF= tetralogy of Fallot; PA= pulmonary atresia; PS=pulmonary stenosis; VSD=ventricular septal defect.

-data are not shown (the sample size was less than 10).

|  | **Number of patients** | | | | |
| --- | --- | --- | --- | --- | --- |
|  | **Total** | **Pre-pandemic baseline** | **Transition period** | **Restriction period** | **Post restriction period** |
| **The whole cohort** | 4900 | 1545 | 1175 | 1375 | 810 |
| **By CHD diagnosis** | | | | | |
| **Functionally single ventricle CHDs** | | | | | |
| **HLHS** | 195 | 60 | 50 | 45 | 40 |
| **FUH (total)** | 180 | 55 | 35 | 60 | 30 |
| Double inlet ventricle | 85 | 30 | 15 | 25 | 10 |
| Tricuspid atresia | 95 | 20 | 20 | 35 | 20 |
| **CHDs where a range of anatomy occurs** | | | | | |
| **TGA (Total)** | 660 | 200 | 150 | 195 | 115 |
| Complex TGA with PS | 60 | 20 | 15 | 15 | - |
| Complex TGA without PS | 240 | 65 | 55 | 75 | 45 |
| TGA with intact ventricular septum | 360 | 115 | 85 | 105 | 60 |
| **PA (Total)** | 290 | 85 | 70 | 75 | 60 |
| PA with VSD | 195 | 60 | 50 | 50 | 35 |
| PA with intact ventricular septum | 95 | 25 | 15 | 30 | 25 |
| **AVSD (Total)** | 590 | 190 | 135 | 215 | 100 |
| Tetralogy AVSD | 35 | 15 | - | 10 | - |
| Unbalanced AVSD | 60 | 20 | 15 | 15 | - |
| Complete AVSD | 500 | 155 | 110 | 150 | 80 |
| **Biventricular CHDs** | | | | | |
| **TOF (Total)** | 820 | 260 | 200 | 225 | 135 |
| Tetralogy absent pulmonary valve | 20 | - | - | - | - |
| Tetralogy with DORV | 110 | 25 | 30 | 35 | 20 |
| Standard tetralogy | 690 | 225 | 170 | 185 | 110 |
| **AOS** | 225 | 95 | 40 | 65 | 30 |
| AOS with muti-level left heart obstruction | 65 | 30 | - | 20 | - |
| Isolated AOS | 160 | 65 | 30 | 45 | 20 |
| **COA (Total)** | 740 | 220 | 195 | 205 | 120 |
| COA with VSD | 280 | 85 | 80 | 65 | 45 |
| Isolated COA | 465 | 135 | 115 | 140 | 75 |
| **VSD (Total)** | 1200 | 380 | 300 | 325 | 195 |
| Multiple VSDs | 95 | 35 | 25 | 20 | 15 |
| Single VSD | 1105 | 345 | 275 | 305 | 180 |

## Supplementary Table S3: Age of pathway procedures by birth era (measured in days since birth).

Data are n(%) or median (IQR).

There were 15 patients who had both a reparative procedure and a single ventricle stage 2 (CHD subgroups: PA, AVSD, and TOF), and their first occurring procedures were included. P-values for the Wilcoxon rank sum test to determine statistical evidence for a delay in procedure timing between each pandemic period compared to the pre-pandemic baseline period are listed in Table S4.

AOS=congenital aortic stenosis; AVSD=atrioventricular septal defect; COA= coarctation of the aorta; DORV=double outlet right ventricle; FUH=functionally univentricular heart; HLHS=hypoplastic left heart syndrome; TGA=transposition of the great arteries; TOF= tetralogy of Fallot; PA= pulmonary atresia; PS=pulmonary stenosis; VSD=ventricular septal defect.

-Results are not shown (number of patients who had pathway procedure was less than 10).

|  | **Total** | | **Pre-pandemic baseline** | | **Transition period** | | **Restriction period** | | **Post restriction period** | |
| --- | --- | --- | --- | --- | --- | --- | --- | --- | --- | --- |
|  | **n (%)** | **Age at operation** | **n (%)** | **Age at operation** | **n (%)** | **Age at operation** | **n (%)** | **Age at operation** | **n (%)** | **Age at operation** |
| ***Palliative stage 1 procedure*** | | | | | | | | | | |
| **The whole cohort** | 1010 (20.6%) | 32 (16, 71) | 315 (20.3%) | 34 (16, 73) | 255 (21.9%) | 34 (16, 70) | 280 (20.3%) | 29 (15, 64) | 160 (19.7%) | 27 (16, 76) |
| ***Palliative stage 2 and reparative procedure*** | | | | | | | | | | |
| **The whole cohort** | 4535 (92.6%) | 131 (30, 217) | 1430 (92.6%) | 139 (32, 227) | 1090 (92.9%) | 124 (30, 214) | 1275 (92.8%) | 126 (31, 215) | 740 (91.5%) | 135 (26, 208) |
| **By CHD diagnosis** | | | | | | | | | | |
| ***Palliative stage 1 procedure*** | | | | | | | | | | |
| **HLHS** | 190 (98.4%) | 13 (8, 18) | 55 (98.3%) | 13 (8, 18) | 50 (98.0%) | 14 (8, 17) | 45 (100%) | 12 (8, 17) | 35 (97.4%) | 14 (9, 20) |
| **FUH (total)** | 160 (87.8%) | 21 (14, 42) | 45 (85.2%) | 20 (12, 44) | 35 (89.2%) | 21 (15, 44) | 50 (86.7%) | 22 (14, 36) | 30 (93.3%) | 22 (17, 45) |
| Double inlet ventricle | 75 (88.1%) | 20 (13, 44) | 30 (87.5%) | 22 (13, 54) | 15 (87.5%) | 18 (12, 27) | 20 (87.5%) | 19 (14, 25) | 10 (91.7%) | 22 (16, 46) |
| Tricuspid atresia | 85 (87.6%) | 25 (16, 40) | 20 (81.8%) | 19 (13, 31) | 20 (90.5%) | 28 (19, 49) | 30 (86.1%) | 25 (14, 37) | 15 (94.4%) | 23 (17, 41) |
| **TGA (Total)** | 55 (8.6%) | 28 (20, 55) | 20 (8.9%) | 52 (26, 77) | 15 (8.6%) | 39 (25, 45) | - | - | 15 (14.9%) | 21 (16, 47) |
| Complex TGA with PA | 30 (53.3%) | 28 (20, 55) | 10 (52.4%) | 49 (22, 60) | - | - | - | - | - | - |
| Complex TGA without PS | 25 (10.0%) | 27 (20, 60) | - | - | - | - | - | - | - | - |
| TGA with intact ventricular septum | - | - | - | - | - | - | - | - | - | - |
| **PA (Total)** | 110 (38.4%) | 24 (16, 53) | 40 (43.7%) | 22 (17, 45) | 25 (36.8%) | 29 (15, 64) | 30 (39.5%) | 25 (17, 52) | 20 (31.0%) | 24 (14, 74) |
| PA with VSD | 80 (42.1%) | 28 (15, 59) | 30 (46.7%) | 25 (16, 50) | 20 (40.4%) | 30 (15, 64) | 20 (41.7%) | 40 (17, 58) | 15 (37.1%) | 25 (15, 89) |
| PA with intact ventricular septum | 30 (30.9%) | 22 (17, 30) | 10 (37.0%) | 21 (18, 29) | - | - | 10 (35.7%) | 21 (17, 26) | - | - |
| **AVSD (Total)** | 140 (24.0%) | 46 (20, 88) | 45 (23.3%) | 46 (18, 80) | 35 (27.8%) | 45 (23, 81) | 50 (27.4%) | 43 (21, 83) | 10 (13.3%) | 104 (62, 137) |
| Tetralogy AVSD | 20 (51.4%) | 68 (32, 123) | - | - | - | - | - | - | - | - |
| Unbalanced AVSD | 35 (56.9%) | 37 (15, 94) | - | - | 10 (73.3%) | 45 (18, 120) | 10 (75.0%) | 38 (15, 93) | - | - |
| Complete AVSD | 90 (18.3%) | 50 (20, 82) | 30 (18.2%) | 46 (16, 76) | 25 (20.7%) | 46 (22, 74) | 30 (19.1%) | 50 (22, 83) | 10 (13.6%) | 113 (50, 140) |
| **TOF (Total)** | 135 (16.3%) | 39 (24, 67) | 35 (13.5%) | 41 (28, 70) | 40 (19.4%) | 37 (26, 60) | 40 (18.2%) | 38 (24, 57) | 20 (14.1%) | 45 (24, 88) |
| Tetralogy absent pulmonary valve | - | - | - | - | - | - | - | - | - | - |
| Tetralogy with DORV | 35 (31.2%) | 38 (25, 82) | - | - | 10 (35.7%) | 32 (24, 49) | 15 (41.2%) | 54 (26, 76) | - | - |
| Standard tetralogy | 100 (14.3%) | 39 (24, 65) | 25 (11.9%) | 40 (28, 66) | 30 (17.3%) | 38 (26, 66) | 25 (14.4%) | 32 (24, 56) | 15 (14.7%) | 49 (25, 87) |
| **AOS (Total)** | 25 (10.2%) | 27 (18, 86) | 15 (14.0%) | 37 (18, 84) | - | - | - | - | - | - |
| AOS with muti- level left heart obstruction | 25 (34.3%) | 27 (18, 86) | 15 (44.8%) | 37 (18, 84) | - | - | - | - | - | - |
| Isolated AOS | - | - | - | - | - | - | - | - | - | - |
| **COA (Total)** | - | - | - | - | - | - | - | - | - | - |
| COA with VSD | - | - | - | - | - | - | - | - | - | - |
| Isolated COA | - | - | - | - | - | - | - | - | - | - |
| **VSD (Total)** | 190 (15.8%) | 91 (61, 164) | 60 (15.5%) | 93 (66, 160) | 60 (19.4%) | 82 (57, 166) | 45 (13.8%) | 108 (64, 228) | 25 (13.8%) | 98 (60, 159) |
| Multiple VSDs | 55 (56.4%) | 90 (59, 142) | 15 (50.0%) | 89 (48, 124) | 15 (57.7%) | 90 (64, 146) | 10 (57.9%) | 66 (50, 141) | 10 (66.7%) | 120 (76, 205) |
| Single VSD | 135 (12.3%) | 92 (61, 170) | 40 (12.1%) | 94 (72, 188) | 45 (15.8%) | 79 (56, 171) | 35 (11.1%) | 112 (65, 238) | 15 (9.4%) | 85 (55, 142) |
| ***Stage 2 and reparative procedure*** | | | | | | | | | | |
| **HLHS** | 140 (72.5%) | 163 (141, 192) | 40 (65.5%) | 166 (149, 187) | 40 (76.5%) | 158 (133, 170) | 35 (78.3%) | 165 (144, 204) | 25 (71.1%) | 171 (144, 229) |
| **FUH (total)** | 165 (90.1%) | 193 (147, 254) | 55 (98.1%) | 198 (145, 248) | 35 (89.2%) | 193 (159, 232) | 55 (90.0%) | 188 (150, 263) | 25 (76.7%) | 180 (154, 264) |
| Double inlet ventricle | 75 (91.7%) | 186 (145, 264) | 30 (96.9%) | 198 (155, 282) | 15 (87.5%) | 180 (160, 239) | 20 (87.5%) | 186 (140, 240) | 10 (91.7%) | 178 (148, 262) |
| Tricuspid atresia | 85 (88.7%) | 198 (150, 249) | 20 (100%) | 191 (134, 236) | 20 (90.5%) | 202 (148, 232) | 35 (91.7%) | 191 (155, 263) | 10 (66.7%) | 213 (167, 265) |
| **TGA (Total)** | 635 (96.1%) | 18 (14, 25) | 195 (95.5%) | 19 (14, 24) | 145 (96.0%) | 18 (14, 25) | 190 (99.0%) | 18 (13, 26) | 105 (92.1%) | 17 (13, 23) |
| Complex TGA & PS | 50 (83.3%) | 215 (65, 378) | 15 (81.0%) | 249 (64, 576) | 15 (86.7%) | 208 (32, 323) | 15 (100%) | 178 (70, 424) | - | - |
| Complex TGA without PS | 230 (95.8%) | 20 (14, 26) | 60 (95.4%) | 21 (15, 24) | 50 (96.2%) | 18 (16, 26) | 75 (98.6%) | 19 (13, 27) | 45 (91.5%) | 20 (14, 30) |
| TGA with intact ventricular septum | 355 (98.3%) | 17 (13, 21) | 115 (98.3%) | 17 (13, 22) | 80 (97.6%) | 17 (12, 22) | 105 (99.0%) | 16 (13, 22) | 55 (98.3%) | 16 (13, 20) |
| **PA (Total)** | 265 (91.0%) | 82 (18, 238) | 75 (88.5%) | 60 (18, 219) | 65 (92.6%) | 97 (22, 266) | 70 (94.7%) | 99 (23, 249) | 50 (87.9%) | 21 (12, 196) |
| PA with VSD | 185 (94.4%) | 128 (34, 280) | 55 (90.0%) | 79 (30, 240) | 50 (98.1%) | 150 (44, 333) | 45 (97.9%) | 204 (79, 292) | 30 (91.4%) | 98 (18, 260) |
| PA with intact ventricular septum | 80 (84.0%) | 16 (10, 102) | 25 (85.2%) | 19 (10, 175) | 10 (75.0%) | 16 (10, 38) | 25 (89.3%) | 19 (13, 101) | 20 (82.6%) | 14 (8, 18) |
| **AVSD (Total)** | 540 (91.7%) | 182 (137, 254) | 180 (94.2%) | 169 (134, 246) | 120 (91.0%) | 185 (138, 252) | 160 (89.4%) | 191 (132, 280) | 85 (92.2%) | 189 (157, 223) |
| Tetralogy AVSD | 35 (97.1%) | 328 (205, 530) | 15 (100%) | 482 (288, 713) | - | - | 10 (90.9%) | 354 (256, 530) | - | - |
| Unbalanced AVSD | 45 (81.0%) | 201 (142, 325) | 15 (81.0%) | 180 (142, 260) | 15 (86.7%) | 201 (158, 259) | 10 (75.0%) | 364 (254, 463) | - | - |
| Complete AVSD | 460 (92.6%) | 174 (132, 226) | 145 (95.5%) | 165 (130, 215) | 100 (91.0%) | 181 (138, 252) | 140 (90.8%) | 178 (128, 230) | 75 (92.6%) | 189 (160, 220) |
| **TOF (Total)** | 805 (98.0%) | 198 (146, 264) | 255 (99.2%) | 206 (147, 290) | 195 (98.0%) | 185 (135, 242) | 220 (97.3%) | 189 (140, 251) | 130 (97.0%) | 219 (166, 276) |
| Tetralogy absent pulmonary valve | 20 (100%) | 115 (58, 238) | - | - | - | - | - | - | - | - |
| Tetralogy with DORV | 100 (92.7%) | 209 (140, 296) | 25 (96.2%) | 224 (121, 337) | 25 (96.4%) | 139 (90, 236) | 30 (91.2%) | 244 (166, 329) | 20 (85.7%) | 220 (170, 272) |
| Standard tetralogy | 680 (98.8%) | 198 (148, 261) | 225 (99.6%) | 204 (148, 278) | 165 (98.2%) | 186 (147, 246) | 185 (98.4%) | 188 (135, 243) | 110 (99.1%) | 217 (168, 276) |
| **AOS (Total)** | 205 (91.1%) | 63 (19, 168) | 85 (89.2%) | 63 (19, 176) | 40 (100%) | 99 (22, 244) | 55 (87.7%) | 44 (18, 141) | 25 (92.9%) | 68 (26, 102) |
| AOS with muti- level left heart obstruction | 50 (76.1%) | 62 (18, 144) | 20 (72.4%) | 97 (33, 150) | - | - | 15 (71.4%) | 44 (14, 85) | - | - |
| Isolated AOS | 155 (97.5%) | 64 (20, 174) | 60 (96.9%) | 54 (19, 182) | 30 (100%) | 71 (22, 219) | 40 (95.5%) | 49 (22, 156) | 20 (100%) | 82 (31, 103) |
| **COA (Total)** | 730 (98.5%) | 22 (14, 54) | 215 (96.4%) | 24 (15, 74) | 195 (99.5%) | 20 (13, 47) | 205 (100%) | 23 (14, 56) | 115 (98.3%) | 21 (15, 35) |
| COA with VSD | 275 (98.2%) | 22 (14, 49) | 80 (96.5%) | 23 (15, 54) | 80 (100%) | 20 (12, 48) | 65 (100%) | 21 (13, 43) | 45 (95.7%) | 22 (17, 45) |
| Isolated COA | 455 (98.7%) | 22 (14, 59) | 130 (96.4%) | 24 (15, 90) | 115 (99.1%) | 20 (13, 47) | 140 (100%) | 25 (14, 67) | 75 (100%) | 20 (15, 34) |
| **VSD (Total)** | 1055 (87.8%) | 156 (107, 244) | 335 (88.7%) | 168 (118, 263) | 260 (87.0%) | 151 (100, 274) | 280 (86.5%) | 154 (105, 234) | 175 (89.8%) | 150 (111, 212) |
| Multiple VSDs | 60 (64.9%) | 204 (115, 495) | 25 (73.5%) | 245 (115, 647) | 20 (69.2%) | 207 (122, 688) | - | - | - | - |
| Single VSD | 995 (89.8%) | 154 (107, 238) | 310 (90.2%) | 166 (119, 244) | 240 (88.6%) | 149 (98, 257) | 270 (88.9%) | 152 (105, 233) | 165 (92.3%) | 148 (108, 207) |

## Supplementary Table S4: P-values for the Wilcoxon rank sum test to determine statistical evidence for a delay in procedure timing between each pandemic period compared to the pre-pandemic baseline period.

Results for age at palliative stage 1 in TGA, AOS and COA was not shown due to limited sample size when broken down by era (n<10) There were 15 patients who had both a reparative procedure and a single ventricle stage 2 (CHD subgroups: PA, AVSD, and TOF), and their first occurring procedures were used.

^The result found in AVSD was based on small sample size (n=10)

AOS=congenital aortic stenosis; AVSD=atrioventricular septal defect; COA= coarctation of the aorta; FUH=functionally univentricular heart; HLHS=hypoplastic left heart syndrome; TGA=transposition of the great arteries; TOF= tetralogy of Fallot; PA= pulmonary atresia; VSD=ventricular septal defect.

|  | **Pre-pandemic baseline** | **Transition period** | **Restriction period** | **Post restriction period** |
| --- | --- | --- | --- | --- |
| ***Age at palliative stage 1 procedure*** | | | | |
| **The whole cohort** | reference | p=0.39 | p=0.75 | p=0.56 |
| **By CHD diagnosis** | | | | |
| **HLHS** | reference | p=0.54 | p=0.59 | p=0.15 |
| **FUH** | reference | p=0.25 | p=0.54 | p=0.21 |
| **PA** | reference | p=0.25 | p=0.32 | p=0.39 |
| **AVSD** | reference | p=0.44 | p=0.44 | p=0.01^ |
| **TOF** | reference | p=0.83 | p=0.74 | p=0.34 |
| **VSD** | reference | p=0.79 | p=0.44 | p=0.58 |
| ***Age at palliative stage 2 and reparative procedure*** | | | | |
| **The whole cohort** | reference | p=0.99 | p=0.98 | p=0.99 |
| **By CHD diagnosis** | | | | |
| **HLHS** | reference | p=0.58 | p=0.52 | p=0.43 |
| **FUH** | reference | p=0.55 | p=0.73 | p=0.83 |
| **TGA** | reference | p=0.07 | p=0.06 | p=0.91 |
| **PA** | reference | p=0.21 | p=0.14 | p=0.09 |
| **AVSD** | reference | p=0.26 | p=0.78 | p=0.69 |
| **AOS** | reference | p=0.99 | p=0.79 | p=0.94 |
| **COA** | reference | p=0.98 | p=0.95 | p=0.18 |
| **TOF** | reference | p=0.96 | p=1 | p=0.99 |
| **VSD** | reference | p=0.99 | p=0.98 | p=0.99 |

## Supplementary Table S5: Univariable and multivariable quantile regression results for median age of pathway procedures (measured in days since birth).

Complete case analysis was performed.

AOS=congenital aortic stenosis; AVSD=atrioventricular septal defect; COA= coarctation of the aorta; DORV=double outlet right ventricle; FUH=functionally univentricular heart; HLHS=hypoplastic left heart syndrome; TGA=transposition of the great arteries; TOF= tetralogy of Fallot; PA= pulmonary atresia; PS=pulmonary stenosis; VSD=ventricular septal defect.

| **Regression results for median age of palliative stage 1 procedure.**  Stage 1 for COA with VSD, TGA with intact ventricular septum and tetralogy absent pulmonary valve were not included in the model due to limited occurrence (n<10 in total). | | | |
| --- | --- | --- | --- |
| **Factor** | **Number of patients (%) n=1000** | **Relative median days with 95% CI**  **(univariate estimates)** | **Relative median days with 95% CI**  **(adjusted estimates)** |
| **Birth era** |  |  |  |
| Pre-pandemic baseline | 310 (30.8%) | Reference | Reference |
| Transition period | 255 (25.5%) | 1 (-11, 12) | -1 (-6, 4) |
| Restriction period | 280 (27.8%) | -4 (-15, 7) | -2 (-7, 2) |
| Post restriction period | 160 (15.9%) | -6 (-20, 8) | 1 (-5, 7) |
| **Ethnic group** |  |  |  |
| White | 720 (71.7%) | Reference | Reference |
| Black | 50 (4.9%) | 12 (-4, 28) | -1 (-11, 10) |
| Asian | 160 (15.9%) | 15 (6, 24)** | 6 (0, 12)* |
| Mixed / Other | 65 (6.5%) | 19 (5, 33)** | 1 (-6, 8) |
| Missing | 10 (1.1%) | N/A | N/A |
| **Deprivation** |  |  |  |
| Quintile 1 (most deprived) | 350 (35.7%) | 6 (-16, 27) | 1 (-5, 7) |
| Quintile 2 | 235 (23.4%) | 5 (-16, 26) | -1 (-7, 6) |
| Quintile 3 | 170 (17.1%) | 6 (-15, 26) | 2 (-4, 8) |
| Quintile 4 | 135 (13.5%) | 7 (-14, 28) | 2 (-7, 10) |
| Quintile 5 (least deprived) | 95 (9.5%) | Reference | Reference |
| Missing | 10 (0.9%) | N/A | N/A |
| **Gender** |  |  |  |
| Female | 460 (45.9%) | Reference | Reference |
| Male | 545 (54.1%) | -14 (-21, -7)*** | -2 (-6, 2) |
| **CHD subgroups** |  |  |  |
| HLHS | 190 (18.9%) | -26 (-32, -20)*** | -24 (-32, -17)*** |
| Double inlet ventricle | 75 (7.4%) | -19 (-26, -12)*** | -17 (-26, -9)*** |
| Tricuspid atresia | 85 (8.5%) | -14 (-21, -7)*** | -15 (-24, -7)*** |
| Complex TGA with PS | 30 (3.2%) | -10 (-29, 8) | -8 (-26, 10) |
| Complex TGA without PS | 25 (2.4%) | -12 (-29, 6) | -10 (-26, 6) |
| PA with VSD | 80 (8.2%) | -11 (-26, 3) | -11 (-25, 3) |
| PA with intact ventricular septum | 30 (2.9%) | -17 (-26, -8)*** | -16 (-25, -8)*** |
| Tetralogy AVSD | 20 (1.8%) | 29 (-12, 71) | 24 (-22, 70) |
| Unbalanced AVSD | 35 (3.3%) | -2 (-23, 19) | -13 (-47, 20) |
| Complete AVSD | 90 (9.1%) | 11 (-2, 24) | 6 (-9, 22) |
| Tetralogy with DORV | 35 (3.4%) | 3 (-22, 27) | -1 (-20, 17) |
| Standard tetralogy | 100 (9.9%) | Reference | Reference |
| AOS with muti-level left heart obstruction | 25 (2.3%) | -12 (-76, 52) | -7 (-67, 52) |
| Multiple VSDs | 55 (5.3%) | 51 (28, 74)*** | 53 (28, 79)*** |
| Single VSD | 135 (13.6%) | 54 (37, 70)*** | 45 (29, 61)*** |
| **Congenital noncardiac comorbidity** | 330 (32.7%) | 24 (16, 32)*** | 3 (-3, 10) |
| **Preterm birth** | 200 (20.0%) | 36 (29, 43)*** | 16 (6, 25)*** |
| **Regression results for median age of single ventricle stage 2 or reparative procedure.** Regression results for median age of single ventricle stage 2 or reparative procedure. There were 15 patients who had both a reparative procedure and a single ventricle stage 2 (CHD subgroups: PA with intact ventricular septum, unbalanced AVSD, tetralogy AVSD, and standard tetralogy), and their first occurring procedures were included in the model. | | | |
| **Factor** | **Number of patients (%) n=4,540** | **Relative median days with 95% CI**  **(univariate estimates)** | **Relative median days with 95% CI**  **(adjusted estimates)** |
| **Birth era** |  |  |  |
| Pre-pandemic baseline | 1430 (31.5%) | Reference | Reference |
| Transition period | 1090 (24.1%) | -15 (-28, -2)* | -3 (-6, 0)* |
| Restriction period | 1275 (28.1%) | -13 (-27, 1)* | -2 (-5, 1) |
| Post restriction period | 740 (16.3%) | -4 (-20, 12) | -1 (-4, 2) |
| **Ethnic group** |  |  |  |
| White | 3310 (73.0%) | Reference | Reference |
| Black | 200 (4.4%) | 14 (-11, 39) | -1 (-10, 8) |
| Asian | 615 (13.6%) | 14 (-2, 30) | 3 (-3, 9) |
| Mixed / Other | 360 (7.9%) | 0 (-18, 18) | -1 (-4, 3) |
| Missing | 50 (1.1%) | N/A | N/A |
| **Deprivation** |  |  |  |
| Quintile 1 most deprived | 1300 (28.7%) | 23 (8, 38)** | 2 (-1, 6) |
| Quintile 2 | 1025 (22.6%) | 11 (-6, 28) | -1 (-4, 3) |
| Quintile 3 | 860 (19.0%) | 11 (-7, 29) | 1 (-3, 4) |
| Quintile 4 | 705 (15.6%) | 9 (-8, 26) | -1 (-5, 3) |
| Quintile 5 (least deprived) | 615 (13.6%) | Reference | Reference |
| Missing | 25 (0.5%) | N/A | N/A |
| **Gender** |  |  |  |
| Female | 1900 (41.9%) | Reference | Reference |
| Male | 2635 (58.1%) | -21 (-32, -11)*** | -1 (-4, 1) |
| **CHD subgroups** |  |  |  |
| HLHS | 140 (3.1%) | -35 (-46, -24)*** | -30 (-42, -19)*** |
| Double inlet ventricle | 75 (1.7%) | -12 (-38, 15) | -7 (-33, 19) |
| Tricuspid atresia | 85 (1.9%) | 0 (-26, 26) | -1 (-25, 24) |
| Complex TGA with PS | 50 (1.1%) | 18 (-63, 98) | -2 (-100, 95) |
| Complex TGA without PS | 230 (5.0%) | -178 (-188, -170)*** | -176 (-186, -166)*** |
| TGA with intact ventricular septum | 355 (7.8%) | -181 (-189, -172)*** | -179 (-189, -169)*** |
| PA with VSD | 60 (1.4%) | -67 (-123, -11)** | -74 (-136, -12)** |
| PA with intact ventricular septum | 185 (4.1%) | -182 (-193, -171)*** | -178 (-189, -167)*** |
| Tetralogy AVSD | 80 (1.7%) | 132 (1, 264)* | 144 (-3, 291)* |
| Unbalanced AVSD | 45 (1.0%) | 3 (-60, 66) | 8 (-55, 70) |
| Complete AVSD | 460 (10.2%) | -24 (-36, -12)*** | -23 (-36, -11)*** |
| Tetralogy absent pulmonary valve | 20 (0.5%) | -83 (-173, 7)* | -104 (-180, -27)** |
| Tetralogy with DORV | 100 (2.2%) | 11 (-21, 44) | 7 (-15, 29) |
| Standard tetralogy | 680 (15.2%) | Reference | Reference |
| AOS with muti-level left heart obstruction | 50 (1.1%) | -136 (-175, -96)*** | -138 (-185, -91)*** |
| Isolated AOS | 155 (3.4%) | -134 (-159, -109)*** | -131 (-155, -107)*** |
| COA with VSD | 275 (6.1%) | -176 (-185, -167)*** | -175 (-184, -166)*** |
| Isolated COA | 455 (10.2%) | -176 (-185, -166)*** | -174 (-183, -165)*** |
| Multiple VSDs | 60 (1.4%) | 6 (-63, 75) | 11 (-79, 101) |
| Single VSD | 995 (22.2%) | -44 (-55, -32)*** | -44 (-56, -32)*** |
| **Congenital noncardiac comorbidity** | 1305 (28.7%) | 51 (41, 61)*** | 0 (-5, 5) |
| **Preterm birth** | 595 (13.1%) | 27 (10, 44)*** | 21 (13, 29)*** |

Significance level (p-value): 0.05 * 0.01 ** 0.001 ***

## Supplementary Table S6: Mortality rate at 1-year (using Kaplan-Meier) with 95% confidence interval by CHD diagnosis subtype and birth era.

AOS=congenital aortic stenosis; AVSD=atrioventricular septal defect; COA= coarctation of the aorta; DORV=double outlet right ventricle; FUH=functionally univentricular heart; HLHS=hypoplastic left heart syndrome; TGA=transposition of the great arteries; TOF= tetralogy of Fallot; PA= pulmonary atresia; PS=pulmonary stenosis; VSD=ventricular septal defect.

-Results are not shown (sample size was less than 10).

| **Diagnosis** | **Mortality rate at 1-year with 95% confidence interval** | | | | |
| --- | --- | --- | --- | --- | --- |
|  | **Total** | **Pre-pandemic baseline** | **Transition period** | **Restriction period** | **Post restriction period** |
| **The whole cohort** | 4.6% (4.0%, 5.2%) | 4.2% (3.2%, 5.2%) | 6.0% (4.6%, 7.3%) | 4.0% (3.0%, 5.0%) | 4.5% (3.0%, 5.9%) |
| ***By CHD diagnosis*** | | | | | |
| **HLHS** | 28.0% (21.4%, 34.0%) | 29.3% (16.6%, 40.1%) | 27.5% (14.1%, 38.7%) | 26.1% (12.2%, 37.7%) | 28.9% (13.0%, 42.0%) |
| **FUH (total)** | 5.5% (2.1%, 8.8%) | 0% | 18.9% (5.3%, 30.6%) | 1.7% (0%, 4.9%) | 6.7% (0%, 15.2%) |
| Double inlet ventricle | 4.8% (0.1%, 9.2%) | 0% | 18.8% (0%, 35.8%) | 0% | 8.3% (0%, 22.7%) |
| Tricuspid atresia | 6.2% (1.3%, 10.9%) | 0% | 19.0% (0.4%, 34.2%) | 2.8% (0%, 8.0%) | 5.6% (0%, 15.6%) |
| **TGA (Total)** | 5.9% (4.1%, 7.7%) | 4.0% (1.2%, 6.6%) | 8.6% (4.0%, 13.0%) | 6.2% (2.7%, 9.5%) | 5.3% (1.1%, 9.3%) |
| Complex TGA with PS | 8.3% (1.1%, 15.1%) | 9.5% (0%, 21.2%) | 20.0% (0%, 37.9%) | 0% | 0% |
| Complex TGA without PS | 9.6% (5.8%, 13.3%) | 4.6% (0%, 9.6%) | 11.3% (2.4%, 19.5%) | 13.5% (5.4%, 21.0%) | 8.5% (0.2%, 16.2%) |
| TGA with intact ventricular septum | 3.0% (1.3%, 4.8%) | 2.6% (0%, 5.4%) | 4.8% (0.1%, 9.3%) | 1.9% (0%, 4.5%) | 3.4% (0%, 8.0%) |
| **PA (Total)** | 7.6% (4.5%, 10.6%) | 6.9% (1.4%, 12.1%) | 10.3% (2.8%, 17.2%) | 6.6% (0.8%, 12.0%) | 6.9% (0.1%, 13.2%) |
| PA with VSD | 6.2% (2.7%, 9.5%) | 6.7% (0.1%, 12.8%) | 7.7% (0.2%, 14.7%) | 4.2% (0%, 9.7%) | 5.7% (0%, 13.1%) |
| PA with intact ventricular septum | 10.6% (4.2%, 16.7%) | 7.4% (0%, 16.8%) | 18.8% (0%, 35.8%) | 10.7% (0%, 21.5%) | 8.7% (0%, 19.5%) |
| **AVSD (Total)** | 8.8% (6.5%, 11.1%) | 9.0% (4.8%, 13.0%) | 13.5% (7.5%, 19.2%) | 6.1% (2.6%, 9.6%) | 6.7% (1.4%, 11.7%) |
| Tetralogy AVSD | 5.7% (0%, 13.1%) | 7.1% (0%, 19.7%) | - | 0% | 0% |
| Unbalanced AVSD | 15.5% (5.7%, 24.3%) | 23.8% (3.2%, 40.0%) | 13.3% (0%, 28.9%) | 6.2% (0%, 17.4%) | - |
| Complete AVSD | 8.2% (5.8%, 10.6%) | 7.1% (3.0%, 11.1%) | 13.5% (6.9%, 19.6%) | 6.6% (2.6%, 10.4%) | 6.2% (0.8%, 11.3%) |
| **TOF (Total)** | 2.2% (1.2%, 3.2%) | 1.9% (0.2%, 3.6%) | 2.0% (0%, 3.9%) | 2.2% (0.3%, 4.1%) | 3.0% (0.1%, 5.8%) |
| Tetralogy absent pulmonary valve | 9.5% (0%, 21.2%) | - | 0% | 0% | - |
| Tetralogy with DORV | 2.8% (0%, 5.8%) | 3.8% (0%, 11.0%) | 3.6% (0%, 10.2%) | 2.9% (0%, 8.5%) | 0% |
| Standard tetralogy | 1.9% (0.9%, 2.9%) | 1.3% (0%, 2.8%) | 1.8% (0%, 3.8%) | 2.1% (0%, 4.2%) | 2.8% (0%, 5.8%) |
| **AOS (Total)** | 3.6% (1.1%, 5.9%) | 4.3% (0.1%, 8.3%) | 0% | 6.2% (0.1%, 11.8%) | 0% |
| AOS with muti-level left heart obstruction | 7.5% (1.0%, 13.5%) | 6.9% (0%, 15.7%) | 0% | 14.3% (0%, 28.0%) | 0% |
| Isolated AOS | 1.9% (0%, 4.0%) | 3.1% (0%, 7.3%) | 0% | 2.3% (0%, 6.6%) | 0% |
| **COA (Total)** | 1.8% (0.8%, 2.7%) | 2.7% (0.5%, 4.8%) | 0.5% (0%, 1.5%) | 2.4% (0.3%, 4.5%) | 0.8% (0%, 2.5%) |
| COA with VSD | 3.2% (1.1%, 5.3%) | 4.7% (0.1%, 9.1%) | 1.2% (0%, 3.6%) | 4.5% (0%, 9.4%) | 2.2% (0%, 6.3%) |
| Isolated COA | 0.9% (0%, 1.7%) | 1.5% (0%, 3.4%) | 0% | 1.4% (0%, 3.4%) | 0% |
| **VSD (Total)** | 0.8% (0.3%, 1.3%) | 0.5% (0%, 1.3%) | 2.0% (0.4%, 3.6%) | 0% | 1.0% (0%, 2.4%) |
| Multiple VSDs | 1.1% (0%, 3.1%) | 2.9% (0%, 8.5%) | 0% | 0% | 0% |
| Single VSD | 0.8% (0.3%, 1.3%) | 0.3% (0%, 0.9%) | 2.2% (0.4%, 3.9%) | 0% | 1.1% (0%, 2.6%) |

## Supplementary Table S7: Univariable and multivariable logistic regression results for infant mortality (before age of 1 year).

To increase the degrees of freedom, ethnicity, deprivation and CHD diagnosis subgroup were collapsed into broad groups. Complete case analysis was performed.

AOS=congenital aortic stenosis; AVSD=atrioventricular septal defect; COA=coarctation of the aorta; FUH=functionally univentricular heart; HLHS=hypoplastic left heart syndrome; TGA=transposition of the great arteries; TOF= tetralogy of Fallot; PA= pulmonary atresia; VSD=ventricular septal defect.

-Results are not shown (the sample size was greater than 0 and less than 10).

| **Factor** | **Overall number of patients**  **(n=4900)** | **Number of deaths under age one year old (%)**  **(n=225 (4.6%))** | **Univariable odds ratio**  **(95% CI)** | **Adjusted odds ratio (95% CI)** |
| --- | --- | --- | --- | --- |
| **Birth era** |  |  |  |  |
| Pre-pandemic baseline | 1545 | 65 (4.2%) | Reference | Reference |
| Transition period | 1175 | 70 (6.0%) | 1.44 (0.98, 2.11)* | 1.60 (1.06, 2.42)* |
| Restriction period | 1375 | 55 (4.0%) | 0.95 (0.63, 1.42) | 1.01 (0.66, 1.56) |
| Post restriction period | 810 | 35 (4.5%) | 1.06 (0.67, 1.68) | 1.05 (0.64, 1.71) |
| **Ethnicity** |  |  |  |  |
| Non-white | 1270 | 70 (5.4%) | Reference | Reference |
| White | 3570 | 155 (4.3%) | 0.85 (0.62, 1.17) | 0.83 (0.59, 1.18) |
| Missing | 65 | - | N/A | N/A |
| **Deprivation** |  |  |  |  |
| Non-deprived area | 2330 | 75 (3.3%) | Reference | Reference |
| Deprived area | 2545 | 145 (5.7%) | 1.80 (1.33, 2.42)*** | 1.56 (1.11, 2.18)** |
| Missing | 25 | - | N/A | N/A |
| **Gender** |  |  |  |  |
| Female | 2090 | 100 (4.7%) | Reference | Reference |
| Male | 2810 | 125 (4.5%) | 0.95 (0.71, 1.28) | 0.90 (0.66, 1.25) |
| **CHD diagnosis** |  |  |  |  |
| HLHS | 195 | 55 (28.0%) | 17.31 (9.31, 32.20)*** | 21.73 (11.29, 41.85)*** |
| FUH | 180 | 10 (5.5%) | 2.61 (1.09, 6.23)* | 3.07 (1.26, 7.51)** |
| TGA | 660 | 40 (5.9%) | 2.79 (1.49, 5.23)*** | 3.67 (1.89, 7.10)*** |
| PA | 290 | 20 (7.6%) | 3.67 (1.82, 7.42)*** | 3.74 (1.82, 7.70)*** |
| AVSD | 590 | 50 (8.8%) | 4.30 (2.35, 7.86)*** | 3.71 (1.94, 7.10)*** |
| TOF | 820 | 20 (2.2%) | Reference | Reference |
| AOS | 225 | - | 1.64 (0.65, 4.17) | 1.95 (0.72, 5.29) |
| COA | 740 | 15 (1.8%) | 0.79 (0.36, 1.76) | 0.91 (0.40, 2.04) |
| VSD | 1200 | 10 (0.8%) | 0.37 (0.16, 0.88)* | 0.34 (0.14, 0.84)** |
| **Congenital noncardiac comorbidity** | 1430 | 85 (6.0%) | 1.52 (1.12, 2.06)** | 1.39 (0.93, 2.09) |
| **Preterm birth** | 695 | 55 (8.2%) | 2.14 (1.52, 3.02)*** | 2.74 (1.87, 4.02)*** |

Significance level (p-value): 0.05 * 0.01 ** 0.001 ***.

## Supplementary Table S8: P-values for the Wilcoxon rank sum test to determine statistical evidence for difference in length of hospital stay during the first year of patients’ lives between each pandemic period compared to the pre-pandemic baseline period.

AOS=congenital aortic stenosis; AVSD=atrioventricular septal defect; COA= coarctation of the aorta; FUH=functionally univentricular heart; HLHS=hypoplastic left heart syndrome; TGA=transposition of the great arteries; TOF= tetralogy of Fallot; PA= pulmonary atresia; VSD=ventricular septal defect.

|  | **Pre-pandemic baseline** | **Transition period** | **Restriction period** | **Post restriction period** |
| --- | --- | --- | --- | --- |
| **Length of overall hospital stay before the age of 1 year (including inpatient, outpatient and emergency care services)** | | | | |
| **The whole cohort** | reference | p=0.13 | p<0.001 | p<0.001 |
| **By CHD diagnosis** | | | | |
| **HLHS** | reference | p=0.81 | p=0.99 | p=0.01 |
| **FUH** | reference | p=0.24 | p=0.68 | p=0.48 |
| **TGA** | reference | p=0.41 | p<0.001 | p<0.001 |
| **PA** | reference | p=0.36 | p=0.95 | p=0.12 |
| **AVSD** | reference | p=0.33 | p=0.96 | p=0.26 |
| **AOS** | reference | p=0.06 | p<0.001 | p=0.001 |
| **COA** | reference | p=0.93 | p=0.37 | p=0.13 |
| **TOF** | reference | p=0.03 | p=0.08 | p=0.05 |
| **VSD** | reference | p=0.58 | p=0.02 | p=0.003 |
| **Length of inpatient hospital stay** | | | | |
| **The whole cohort** | reference | p=0.41 | p<0.001 | p<0.001 |
| **By CHD diagnosis** | | | | |
| **HLHS** | reference | p=0.72 | p=0.31 | p=0.86 |
| **FUH** | reference | p=0.41 | p=0.004 | p=0.4 |
| **TGA** | reference | p=0.33 | p=0.003 | p=0.002 |
| **PA** | reference | p=0.37 | p=0.001 | p=0.16 |
| **AVSD** | reference | p=0.25 | p<0.001 | p=0.02 |
| **AOS** | reference | p=0.02 | p<0.001 | p<0.001 |
| **COA** | reference | p=0.57 | p=0.11 | p=0.23 |
| **TOF** | reference | p=0.01 | p=0.008 | p=0.008 |
| **VSD** | reference | p=0.02 | p=0.62 | p=0.01 |
| **Length of outpatient hospital stay** | | | | |
| **The whole cohort** | reference | p=0.13 | p=0.004 | p<0.001 |
| **By CHD diagnosis** | | | | |
| **HLHS** | reference | p=0.95 | p=0.43 | p=0.04 |
| **FUH** | reference | p=0.32 | p=0.37 | p=0.57 |
| **TGA** | reference | p=0.29 | p=0.008 | p<0.001 |
| **PA** | reference | p=0.7 | p=0.26 | p=0.35 |
| **AVSD** | reference | p=0.57 | p=0.3 | p=0.33 |
| **AOS** | reference | p=0.38 | p=0.03 | p=0.05 |
| **COA** | reference | p=0.92 | p=0.82 | p=0.52 |
| **TOF** | reference | p=0.04 | p=0.24 | p=0.07 |
| **VSD** | reference | p=0.57 | p=0.04 | p=0.04 |

## Supplementary Table S9: Length of overall hospital stay before the age of 1 year by CHD diagnosis subgroup, ethnicity, and deprivation among birth eras

Data are median days (IQR). Patients from Wales (n=235, 4.8% of the whole cohort) were not included because we don’t have their outpatient and emergency care records. Additionally, patients with missing data ethnicity (n=10) and deprivation (n=25) were not included in the sub tables of ethnicity and deprivation due to limited sample size. P-values for the Wilcoxon rank sum test to determine statistical evidence for a difference in length of hospital stay during the first year of patients’ lives between each pandemic period compared to the pre-pandemic baseline period were listed in Table S8.

Separate inpatient and outpatient data were shown in Supplementary table S10-11. Emergency visit data are not presented separately since these were a median of days 1-2 for all CHD diagnoses.

AOS=congenital aortic stenosis; AVSD=atrioventricular septal defect; COA= coarctation of the aorta; DORV=double outlet right ventricle; FUH=functionally univentricular heart; HLHS=hypoplastic left heart syndrome; TGA=transposition of the great arteries; TOF= tetralogy of Fallot; PA= pulmonary atresia; PS=pulmonary stenosis; VSD=ventricular septal defect.

-Results are not shown (sample size was less than 10).

| **Length of hospital stay (** **measured in days; including inpatient, outpatient and emergency care services) before the age of 1 year** | | | | | |
| --- | --- | --- | --- | --- | --- |
|  | **Total** | **Pre-pandemic baseline** | **Transition period** | **Restriction period** | **Post restriction period** |
| **The whole cohort** | 44 (26, 80) | 40 (24, 76) | 41 (25, 71) | 47 (29, 80) | 50 (31, 92) |
| **By CHD diagnosis** | | | | | |
| **HLHS** | 113 (64, 164) | 109 (53, 148) | 110 (64, 159) | 106 (62, 156) | 150 (87, 222) |
| **FUH (total)** | 80 (59, 118) | 83 (58, 118) | 69 (55, 90) | 80 (65, 116) | 96 (58, 123) |
| Double inlet ventricle | 76 (58, 116) | 74 (56, 118) | 68 (52, 99) | 80 (62, 100) | 92 (64, 128) |
| Tricuspid atresia | 80 (59, 118) | 92 (63, 118) | 69 (58, 86) | 83 (67, 122) | 98 (48, 123) |
| **TGA (Total)** | 33 (25, 50) | 30 (24, 42) | 32 (23, 46) | 36 (27, 54) | 42 (28, 58) |
| Complex TGA with PS | 70 (42, 116) | 47 (39, 99) | 54 (33, 101) | 75 (44, 154) | - |
| Complex TGA without PS | 40 (28, 58) | 34 (27, 46) | 38 (27, 55) | 42 (28, 60) | 46 (32, 68) |
| TGA with intact ventricular septum | 29 (23, 39) | 26 (21, 34) | 28 (21, 36) | 31 (24, 43) | 32 (25, 44) |
| **PA (Total)** | 78 (48, 122) | 78 (45, 105) | 83 (48, 126) | 73 (46, 115) | 87 (61, 146) |
| PA with VSD | 92 (54, 146) | 89 (48, 135) | 94 (46, 146) | 79 (65, 126) | 104 (67, 162) |
| PA with intact ventricular septum | 62 (42, 87) | 53 (38, 82) | 74 (54, 108) | 66 (41, 88) | 62 (40, 86) |
| **AVSD (Total)** | 69 (43, 110) | 68 (41, 124) | 63 (40, 92) | 68 (48, 106) | 75 (48, 118) |
| Tetralogy AVSD | 81 (50, 138) | 70 (43, 141) | - | 94 (81, 151) | - |
| Unbalanced AVSD | 74 (47, 118) | 68 (45, 107) | 73 (58, 123) | 96 (66, 122) | - |
| Complete AVSD | 67 (42, 108) | 67 (39, 124) | 62 (36, 92) | 66 (45, 102) | 79 (51, 119) |
| **TOF (Total)** | 44 (28, 71) | 36 (24, 65) | 43 (28, 66) | 48 (30, 83) | 49 (33, 72) |
| Tetralogy absent pulmonary valve | 182 (79, 263) | - | - | - | - |
| Tetralogy with DORV | 71 (40, 128) | 80 (50, 185) | 55 (41, 142) | 88 (51, 136) | 60 (38, 81) |
| Standard tetralogy | 40 (26, 63) | 32 (23, 59) | 39 (28, 62) | 45 (30, 71) | 48 (32, 70) |
| **AOS (Total)** | 25 (13, 45) | 22 (11, 45) | 23 (16, 34) | 26 (14, 42) | 32 (16, 62) |
| AOS with muti-level left heart obstruction | 40 (26, 80) | 36 (22, 68) | - | 40 (26, 86) | - |
| Isolated AOS | 20 (11, 32) | 19 (10, 30) | 20 (13, 31) | 22 (13, 32) | 21 (14, 38) |
| **COA (Total)** | 31 (20, 51) | 28 (18, 46) | 31 (22, 58) | 33 (20, 55) | 31 (23, 51) |
| COA with VSD | 39 (25, 68) | 36 (25, 58) | 39 (26, 69) | 40 (24, 62) | 37 (28, 82) |
| Isolated COA | 26 (19, 43) | 24 (16, 36) | 26 (20, 43) | 31 (19, 49) | 28 (21, 44) |
| **VSD (Total)** | 41 (26, 72) | 40 (23, 69) | 37 (23, 67) | 45 (29, 72) | 45 (32, 79) |
| Multiple VSDs | 52 (30, 96) | 58 (32, 98) | 38 (24, 87) | 56 (24, 214) | 54 (38, 79) |
| Single VSD | 41 (26, 70) | 38 (22, 65) | 37 (23, 66) | 45 (29, 71) | 44 (31, 78) |
| **By ethnicity** | | | | | |
|  | **Total** | **Pre-pandemic baseline** | **Transition period** | **Restriction period** | **Post restriction period** |
| White | 43 (26, 80) | 39 (24, 73) | 39 (25, 71) | 46 (28, 80) | 47 (30, 84) |
| Black | 57 (30, 102) | 45 (28, 84) | 58 (32, 90) | 64 (30, 98) | 79 (45, 125) |
| Asian | 52 (30, 102) | 40 (26, 86) | 49 (27, 92) | 55 (34, 90) | 63 (34, 116) |
| Mixed / Other | 43 (25, 73) | 40 (20, 86) | 44 (26, 64) | 44 (25, 74) | 45 (30, 70) |
| Missing in ethnicity | 39 (22, 84) | - | - | - | - |
| **By IMD (area deprivation) score** | | | | | |
|  | **Total** | **Pre-pandemic baseline** | **Transition period** | **Restriction period** | **Post restriction period** |
| Quintile 1 (most deprived) | 48 (29, 88) | 44 (27, 87) | 45 (28, 81) | 54 (31, 88) | 55 (32, 108) |
| Quintile 2 | 45 (27, 84) | 40 (25, 81) | 40 (24, 78) | 50 (31, 86) | 50 (30, 88) |
| Quintile 3 | 43 (26, 76) | 36 (23, 70) | 39 (24, 67) | 46 (27, 80) | 52 (32, 105) |
| Quintile 4 | 40 (25, 73) | 36 (22, 70) | 39 (24, 73) | 44 (27, 75) | 44 (30, 68) |
| Quintile 5 (least deprived) | 42 (25, 69) | 36 (23, 66) | 41 (24, 70) | 43 (26, 63) | 46 (31, 77) |
| Missing in deprivation | 29 (16, 56) | - | - | - | - |

## Supplementary Table S10: Length of inpatient hospital stay before the age of 1 year by CHD diagnosis subgroup, ethnicity, and deprivation among birth eras

Data are median days (IQR).

AOS=congenital aortic stenosis; AVSD=atrioventricular septal defect; COA= coarctation of the aorta; DORV=double outlet right ventricle; FUH=functionally univentricular heart; HLHS=hypoplastic left heart syndrome; TGA=transposition of the great arteries; TOF= tetralogy of Fallot; PA= pulmonary atresia; PS=pulmonary stenosis; VSD=ventricular septal defect.

-Results are not shown (sample size was less than 10).

| **Length of inpatient hospital stay (measured in days) before the age of 1 year** | | | | | |
| --- | --- | --- | --- | --- | --- |
|  | **Total** | **Pre-pandemic baseline** | **Transition period** | **Restriction period** | **Post restriction period** |
| **The whole cohort** | 27 (15, 55) | 24 (13, 52) | 26 (15, 54) | 29 (16, 55) | 30 (17,61) |
| **By CHD diagnosis** | | | | | |
| **HLHS** | 86 (43, 145) | 90 (42, 126) | 86 (44, 142) | 62 (36, 127) | 118 (64, 218) |
| **FUH (total)** | 51 (37, 85) | 54 (38, 85) | 45 (35, 65) | 46 (36, 84) | 54 (42, 96) |
| Double inlet ventricle | 51 (37, 84) | 54 (38, 88) | 44 (34, 76) | 43 (34, 78) | 58 (48, 108) |
| Tricuspid atresia | 51 (37, 87) | 56 (39, 84) | 45 (37, 61) | 49 (36, 90) | 54 (38, 96) |
| **TGA (Total)** | 25 (18, 38) | 22 (18, 31) | 25 (17, 38) | 26 (19, 42) | 28 (21, 46) |
| Complex TGA with PS | 51 (29, 86) | 40 (23, 81) | 45 (28, 77) | 54 (31, 106) | - |
| Complex TGA without PS | 29 (21, 47) | 27 (21, 37) | 27 (19, 51) | 32 (20, 51) | 32 (24, 48) |
| TGA with intact ventricular septum | 21 (17, 29) | 20 (16, 26) | 22 (16, 28) | 22 (18, 31) | 24 (18, 30) |
| **PA (Total)** | 49 (29, 94) | 55 (27, 84) | 52 (29, 99) | 44 (29, 66) | 54 (32, 118) |
| PA with VSD | 58 (32, 114) | 65 (32, 106) | 55 (28, 125) | 48 (33, 94) | 81 (46, 139) |
| PA with intact ventricular septum | 34 (23, 56) | 34 (24, 54) | 46 (32, 66) | 34 (23, 49) | 32 (19, 64) |
| **AVSD (Total)** | 44 (22, 81) | 46 (20, 98) | 43 (23, 77) | 43 (22, 76) | 48 (27, 96) |
| Tetralogy AVSD | 56 (17, 80) | 47 (14, 88) | - | 59 (30, 75) | - |
| Unbalanced AVSD | 50 (36, 94) | 46 (30, 71) | 54 (38, 95) | 70 (48, 96) | - |
| Complete AVSD | 43 (22, 80) | 46 (20, 100) | 38 (22, 75) | 38 (21, 66) | 52 (30, 97) |
| **TOF (Total)** | 21 (13, 45) | 20 (12, 40) | 21 (13, 41) | 23 (14, 50) | 26 (15, 48) |
| Tetralogy absent pulmonary valve | 161 (39, 249) | - | - | - | - |
| Tetralogy with DORV | 37 (20, 86) | 44 (20, 118) | 34 (22, 104) | 48 (20, 103) | 28 (23, 49) |
| Standard tetralogy | 20 (13, 38) | 18 (12, 33) | 20 (12, 36) | 21 (13, 42) | 23 (14, 44) |
| **AOS (Total)** | 13 (7, 31) | 12 (7, 33) | 13 (7, 25) | 16 (6, 29) | 14 (6, 37) |
| AOS with muti-level left heart obstruction | 29 (15, 52) | 26 (11, 41) | - | 30 (17, 53) | - |
| Isolated AOS | 10 (5, 21) | 10 (5, 22) | 10 (6, 24) | 9 (5, 20) | 10 (4, 20) |
| **COA (Total)** | 20 (13, 37) | 19 (12, 34) | 21 (14, 38) | 21 (13, 39) | 22 (15, 36) |
| COA with VSD | 26 (18, 48) | 26 (18, 42) | 28 (20, 54) | 26 (16, 47) | 26 (17, 49) |
| Isolated COA | 17 (12, 30) | 16 (10, 24) | 18 (13, 31) | 18 (12, 36) | 19 (13, 29) |
| **VSD (Total)** | 23 (12, 48) | 19 (10, 48) | 21 (11, 47) | 26 (13, 48) | 24 (14, 51) |
| Multiple VSDs | 28 (16, 72) | 40 (17, 77) | 24 (10, 72) | 22 (14, 129) | 28 (21, 44) |
| Single VSD | 22 (12, 47) | 18 (10, 45) | 21 (11, 46) | 26 (13, 48) | 24 (12, 51) |
| **By ethnicity** | | | | | |
|  | **Total** | **Pre-pandemic baseline** | **Transition period** | **Restriction period** | **Post restriction period** |
| White | 26 (15, 51) | 23 (13, 49) | 25 (14, 51) | 28 (16, 52) | 29 (16, 53) |
| Black | 38 (18, 79) | 32 (16, 62) | 40 (17, 76) | 42 (18, 73) | 58 (26, 103) |
| Asian | 32 (18, 68) | 28 (15, 59) | 32 (17, 73) | 33 (20, 60) | 44 (22, 88) |
| Mixed / Other | 26 (14, 54) | 26 (12, 70) | 28 (15, 44) | 24 (14, 53) | 29 (17, 53) |
| Missing in ethnicity | 13 (6, 23) | 29 (7, 80) | 8 (7, 16) | 10 (6, 18) | 18 (10, 23) |
| **By IMD (area deprivation) score** | | | | | |
|  | **Total** | **Pre-pandemic baseline** | **Transition period** | **Restriction period** | **Post restriction period** |
| Quintile 1 (most deprived) | 32 (17, 62) | 29 (16, 61) | 31 (17, 58) | 33 (17, 61) | 34 (19, 81) |
| Quintile 2 | 27 (15, 56) | 24 (13, 52) | 25 (15, 56) | 29 (17, 60) | 30 (16, 58) |
| Quintile 3 | 26 (14, 53) | 22 (12, 44) | 24 (13, 47) | 28 (16, 56) | 35 (17, 72) |
| Quintile 4 | 23 (14, 47) | 22 (13, 47) | 23 (14, 50) | 23 (14, 45) | 25 (17, 44) |
| Quintile 5 (least deprived) | 24 (13, 47) | 20 (12, 42) | 24 (14, 50) | 24 (15, 42) | 28 (14, 53) |
| Missing in deprivation | 29 (14, 45) | - | - | 22 (14, 47) | - |

## Supplementary Table S11: Length of outpatient hospital stay before the age of 1 year by CHD diagnosis subgroup, ethnicity, and deprivation among birth eras

Data are median days (IQR). Patients from Wales (n=235, 4.8% of the whole cohort) were not included because we don’t have their outpatient records. Additionally, patients with missing data ethnicity (n=10) and deprivation (n=25) were not included in the sub tables of ethnicity and deprivation due to limited sample size.

AOS=congenital aortic stenosis; AVSD=atrioventricular septal defect; COA= coarctation of the aorta; DORV=double outlet right ventricle; FUH=functionally univentricular heart; HLHS=hypoplastic left heart syndrome; TGA=transposition of the great arteries; TOF= tetralogy of Fallot; PA= pulmonary atresia; PS=pulmonary stenosis; VSD=ventricular septal defect.

-Results are not shown (sample size was less than 10).

| **Length of outpatient hospital stay (measured in days) before the age of 1 year** | | | | | |
| --- | --- | --- | --- | --- | --- |
|  | **Total** | **Pre-pandemic baseline** | **Transition period** | **Restriction period** | **Post restriction period** |
| **The whole cohort** | 11 (6, 20) | 10 (6, 17) | 10 (6, 18) | 13 (7, 22) | 13 (7, 22) |
| **By CHD diagnosis** | | | | | |
| **HLHS** | 10 (1, 23) | 10 (1, 17) | 10 (0, 21) | 12 (5, 27) | 10 (1, 22) |
| **FUH (total)** | 19 (11, 30) | 14 (10, 26) | 15 (8, 24) | 28 (15, 34) | 18 (13, 29) |
| Double inlet ventricle | 19 (10, 29) | 15 (10, 26) | 15 (8, 24) | 28 (18, 36) | 20 (14, 30) |
| Tricuspid atresia | 18 (12, 31) | 14 (12, 23) | 15 (8, 23) | 27 (15, 32) | 18 (12, 25) |
| **TGA (Total)** | 6 (4, 11) | 6 (4, 9) | 6 (4, 11) | 8 (4, 13) | 8 (4, 15) |
| Complex TGA with PS | 14 (7, 24) | 10 (6, 16) | 12 (3, 18) | 15 (11, 26) | - |
| Complex TGA without PS | 8 (4, 13) | 7 (4, 9) | 8 (4, 12) | 8 (5, 14) | 8 (4, 14) |
| TGA with intact ventricular septum | 5 (4, 9) | 5 (4, 7) | 6 (4, 7) | 6 (4, 10) | 6 (4, 12) |
| **PA (Total)** | 15 (9, 27) | 12 (8, 20) | 15 (9, 22) | 19 (13, 33) | 18 (10, 27) |
| PA with VSD | 16 (10, 27) | 13 (8, 22) | 14 (9, 22) | 22 (14, 34) | 21 (13, 30) |
| PA with intact ventricular septum | 14 (6, 21) | 11 (5, 17) | 16 (12, 21) | 16 (9, 28) | 12 (4, 19) |
| **AVSD (Total)** | 17 (10, 26) | 15 (10, 22) | 14 (7, 24) | 20 (13, 30) | 19 (12, 27) |
| Tetralogy AVSD | 21 (14, 43) | 18 (15, 49) | - | 40 (32, 47) | - |
| Unbalanced AVSD | 18 (8, 26) | 13 (8, 22) | 14 (7, 23) | 24 (17, 28) | - |
| Complete AVSD | 17 (10, 25) | - | - | - | - |
| **TOF (Total)** | 14 (9, 23) | 15 (10, 22) | 14 (8, 25) | 19 (12, 29) | 19 (12, 27) |
| Tetralogy absent pulmonary valve | 10 (2, 18) | 12 (8, 20) | 14 (10, 22) | 17 (12, 25) | 16 (10, 27) |
| Tetralogy with DORV | 17 (12, 33) | - | - | - | - |
| Standard tetralogy | 14 (9, 22) | 17 (9, 31) | 14 (11, 22) | 24 (15, 39) | 16 (11, 32) |
| **AOS (Total)** | 7 (4, 12) | 12 (8, 19) | 14 (10, 21) | 16 (12, 24) | 16 (10, 26) |
| AOS with muti-level left heart obstruction | 9 (6, 13) | 7 (4, 10) | 8 (4, 13) | 8 (5, 13) | 8 (4, 13) |
| Isolated AOS | 7 (4, 11) | 8 (7, 12) | - | 10 (6, 17) | - |
| **COA (Total)** | 7 (4, 12) | 6 (3, 10) | 7 (4, 10) | 8 (5, 11) | 8 (4, 14) |
| COA with VSD | 8 (5, 13) | 6 (4, 10) | 7 (5, 12) | 7 (4, 14) | 7 (5, 13) |
| Isolated COA | 7 (4, 11) | 7 (4, 12) | 8 (5, 13) | 7 (4, 14) | 9 (6, 13) |
| **VSD (Total)** | 12 (8, 20) | 6 (4, 10) | 7 (5, 10) | 7 (5, 14) | 6 (4, 12) |
| Multiple VSDs | 13 (6, 21) | 12 (7, 20) | 10 (7, 17) | 13 (7, 21) | 14 (10, 22) |
| Single VSD | 12 (8, 20) | 14 (8, 23) | 9 (6, 15) | 7 (5, 14) | 19 (13, 22) |
| **By ethnicity** | | | | | |
|  | **Total** | **Pre-pandemic baseline** | **Transition period** | **Restriction period** | **Post restriction period** |
| White | 11 (6, 20) | 10 (6, 18) | 10 (6, 18) | 13 (7, 22) | 13 (8, 22) |
| Black | 12 (6, 18) | 10 (6, 13) | 12 (7, 18) | 15 (6, 23) | 16 (8, 23) |
| Asian | 13 (7, 12) | 11 (6, 20) | 11 (5, 18) | 15 (8, 25) | 13 (8, 22) |
| Mixed / Other | 10 (5, 18) | 9 (4, 14) | 10 (5, 17) | 12 (6, 20) | 9 (4, 18) |
| Missing in ethnicity | 3 (0, 5) | - | - | - | - |
| **By IMD (area deprivation) score** | | | | | |
|  | **Total** | **Pre-pandemic baseline** | **Transition period** | **Restriction period** | **Post restriction period** |
| Quintile 1 (most deprived) | 11 (6, 18) | 10 (5, 16) | 10 (5, 17) | 13 (6, 21) | 12 (7, 22) |
| Quintile 2 | 12 (6, 20) | 11 (6, 18) | 10 (5, 18) | 14 (7, 24) | 14 (7, 21) |
| Quintile 3 | 11 (6, 20) | 10 (6, 17) | 11 (6, 20) | 13 (6, 21) | 13 (7, 22) |
| Quintile 4 | 12 (6, 20) | 10 (6, 18) | 10 (6, 18) | 16 (8, 25) | 13 (7, 21) |
| Quintile 5 (least deprived) | 12 (7, 21) | 11 (6, 19) | 12 (7, 19) | 13 (7, 20) | 14 (8, 24) |
| Missing in deprivation | 2 (1, 5) | - | - | 2 (1, 5) | - |

## Supplementary Table S12: Univariable and multivariable quantile regression results for median days spent at home in the first year of life.

N=225 (4.5%) infant mortality were assigned at 0 days at home as the worst outcome. Complete case analysis was performed.

AOS=congenital aortic stenosis; AVSD=atrioventricular septal defect; COA=coarctation of the aorta; DORV=double outlet right ventricle; FUH=functionally univentricular heart; HLHS=hypoplastic left heart syndrome; TGA=transposition of the great arteries; TOF= tetralogy of Fallot; PA= pulmonary atresia; PS=pulmonary stenosis; VSD=ventricular septal defect.

| **Factor** | **Number of patients (%) (n=4900)** | **Relative median days with 95% CI**  **(univariate estimates)** | **Relative median days with 95% CI**  **(adjusted estimates)** |
| --- | --- | --- | --- |
| **Birth era** |  |  |  |
| Pre-pandemic baseline | 1545 (31.5%) | Reference | Reference |
| Transition period | 1175 (24.0%) | -2 (-5, 1) | -1 (-3, 1) |
| Restriction period | 1375 (28.0%) | -4 (-7, -1)** | -1 (-3, 1) |
| Post restriction period | 810 (16.5%) | -6 (-9, -3)*** | -2 (-4, 0) |
| **Ethnic group** |  |  |  |
| White | 3570 (72.8%) | Reference | Reference |
| Black | 220 (4.5%) | -13 (-24, -2)* | -7 (-12, -1)* |
| Asian | 665 (13.6%) | -7 (-11, -3)*** | -3 (-6, 0)* |
| Mixed / Other | 380 (7.8%) | 0 (-4, 4) | 0 (-2, 2) |
| Missing | 65 (1.3%) | N/A | N/A |
| **Deprivation** |  |  |  |
| Quintile 1 most deprived | 1435 (29.3%) | -9 (-12, -6)*** | -4 (-6, -2)*** |
| Quintile 2 | 1110 (22.6%) | -4 (-7, -1)** | -2 (-4, 0)* |
| Quintile 3 | 925 (18.8%) | -2 (-6, 2) | -3 (-5, -1)** |
| Quintile 4 | 750 (15.3%) | 0 (-3, 3) | -1 (-3, 1) |
| Quintile 5 (least deprived) | 655 (13.3%) | Reference | Reference |
| Missing | 25 (0.6%) | N/A | N/A |
| **Gender** |  |  |  |
| Female | 2090 (42.6%) | Reference | Reference |
| Male | 2810 (57.4%) | 2 (-1, 5) | 1 (0, 2) |
| **CHD subgroup** |  |  |  |
| HLHS | 195 (3.9%) | -120 (-144, -96)*** | -116 (-138, -94)*** |
| Double inlet ventricle | 85 (1.7%) | -35 (-48, -22)*** | -30 (-42, -18)*** |
| Tricuspid atresia | 95 (2.0%) | -33 (-45, -21)*** | -34 (-43, -25)*** |
| Complex TGA with PS | 60 (1.2%) | -36 (-53, -20)*** | -37 (-53, -21)*** |
| Complex TGA without PS | 240 (4.9%) | -10 (-14, -6)*** | -13 (-17, -10)*** |
| TGA with intact ventricular septum | 360 (7.4%) | -2 (-4, 0)* | -5 (-7, -3)*** |
| PA with VSD | 195 (4.0%) | -44 (-56, -32)*** | -33 (-43, -24)*** |
| PA with intact ventricular septum | 95 (1.9%) | -23 (-33, -13)*** | -19 (-27, -12)*** |
| Tetralogy AVSD | 35 (0.7%) | -37 (-62, -12)** | -1 (-29, 26) |
| Unbalanced AVSD | 60 (1.2%) | -51 (-75, -26)*** | -37 (-62, -12)** |
| Complete AVSD | 500 (10.2%) | -24 (-29, -19)*** | -9 (-14, -4)*** |
| Tetralogy absent pulmonary valve | 20 (0.4%) | -152 (-272, -32)** | -115 (-233, 2)* |
| Tetralogy with DORV | 110 (2.2%) | -17 (-28, -6)*** | -9 (-17, -1)* |
| Standard tetralogy | 690 (14.1%) |  | Reference |
| AOS with muti-level left heart obstruction | 65 (1.4%) | -9 (-17, -1)* | -8 (-17, 1)* |
| Isolated AOS | 160 (3.2%) | 10 (8, 12)*** | 5 (3, 8)*** |
| COA with VSD | 280 (5.7%) | -6 (-9, -3)*** | -7 (-11, -4)*** |
| Isolated COA | 465 (9.4%) | 3 (1, 5)*** | 1 (-1, 2) |
| Multiple VSDs | 95 (1.9%) | -8 (-21, 5) | -9 (-17, 0)* |
| Single VSD | 1105 (22.6%) | -2 (-4, 0) | 1 (-1, 3) |
| **Congenital noncardiac comorbidity** | 1430 (29.2%) | -26 (-30, -22)*** | -20 (-24, -16)*** |
| **Preterm birth** | 695 (14.1%) | -50 (-57, -43)*** | -41 (-48, -34)*** |

Significance level (p-value): 0.05 * 0.01 ** 0.001 ***.

## Supplementary Table S13: Length of overall hospital stay before the age of 1 year by CHD diagnosis subgroup and social determinants (ethnicity and deprivation)

Data are median days (IQR). Patients from Wales (n=235, 4.8% of the whole cohort) were not included because we don’t have their outpatient and emergency care records. Additionally, patients with missing data ethnicity (n=10) and deprivation (n=25) were not included due to limited sample size among each CHD diagnosis.

AOS=congenital aortic stenosis; AVSD=atrioventricular septal defect; COA= coarctation of the aorta; DORV=double outlet right ventricle; FUH=functionally univentricular heart; HLHS=hypoplastic left heart syndrome; TGA=transposition of the great arteries; TOF= tetralogy of Fallot; PA= pulmonary atresia; PS=pulmonary stenosis; VSD=ventricular septal defect.

-Results are not shown (sample size was less than 10).

| **Length of hospital stay (measured in days; including inpatient, outpatient and emergency care services) before the age of 1 year** | | | | | | | | | |
| --- | --- | --- | --- | --- | --- | --- | --- | --- | --- |
| **Diagnosis** | **Ethnicity** | | | | **Deprivation** | | | | |
|  | **White** | **Black** | **Asian** | **Mixed / Other** | **Quintile 1 (most deprived)** | **Quintile 2** | **Quintile 3** | **Quintile 4** | **Quintile 5 (least deprived)** |
| **HLHS** | 110 (60, 158) | 134 (72, 247) | 145 (64, 166) | 130 (73, 170) | 128 (64, 173) | 95 (57, 146) | 125 (90, 182) | 113 (84, 168) | 111 (64, 140) |
| **FUH (total)** | 80 (61, 119) | 174 (147, 180) | 75 (58, 106) | 59 (49, 77) | 68 (54, 113) | 92 (68, 126) | 72 (58, 121) | 76 (66, 101) | 90 (69, 109) |
| Double inlet ventricle | 76 (57, 115) | - | 67 (59, 98) | - | 65 (53, 113) | 93 (67, 128) | 76 (62, 131) | - | - |
| Tricuspid atresia | 86 (66, 121) | - | 80 (57, 108) | 57 (46, 66) | 69 (55, 113) | 86 (74, 120) | 70 (58, 119) | 79 (71, 146) | - |
| **TGA (Total)** | 32 (25, 47) | 36 (30, 83) | 40 (26, 74) | 29 (24, 46) | 33 (27, 51) | 37 (26, 55) | 31 (24, 44) | 31 (24, 45) | 32 (26, 48) |
| Complex TGA with PS | 56 (39, 110) | - | 88 (64, 132) | - | 101 (75, 133) | 100 (46, 142) | 83 (38, 102) | 40 (34, 52) | 65 (37, 127) |
| Complex TGA without PS | 38 (27, 54) | 40 (32, 72) | 56 (40, 91) | 40 (32, 49) | 40 (28, 59) | 44 (34, 70) | 40 (30, 54) | 38 (27, 61) | 34 (26, 46) |
| TGA with intact ventricular septum | 29 (23, 39) | 31 (30, 52) | 32 (23, 39) | 24 (20, 30) | 31 (24, 37) | 31 (21, 41) | 27 (21, 34) | 27 (22, 34) | 28 (25, 46) |
| **PA (Total)** | 78 (45, 117) | 73 (60, 83) | 93 (58, 146) | 67 (52, 100) | 74 (46, 110) | 81 (54, 126) | 75 (52, 125) | 99 (54, 159) | 59 (44, 102) |
| PA with VSD | 93 (54, 143) | - | 94 (60, 148) | 67 (56, 110) | 83 (48, 134) | 99 (65, 146) | 97 (60, 128) | 108 (71, 173) | 64 (47, 140) |
| PA with intact ventricular septum | 60 (39, 83) | - | 85 (53, 116) | - | 62 (42, 83) | 65 (47, 96) | 64 (50, 90) | - | 46 (34, 92) |
| **AVSD (Total)** | 67 (42, 105) | 67 (38, 115) | 89 (50, 139) | 65 (43, 108) | 74 (45, 115) | 71 (42, 118) | 76 (48, 110) | 62 (38, 95) | 66 (38, 88) |
| Tetralogy AVSD | 74 (50, 110) | - | 81 (44, 120) | - | 74 (43, 177) | 87 (42, 118) | - | - | - |
| Unbalanced AVSD | 81 (51, 119) | - | - | - | 72 (46, 108) | - | 104 (93, 129) | - | - |
| Complete AVSD | 63 (41, 101) | 66 (38, 115) | 92 (49, 138) | 66 (45, 107) | 75 (45, 115) | 68 (44, 116) | 69 (47, 105) | 60 (38, 95) | 58 (36, 82) |
| **TOF (Total)** | 45 (27, 70) | 49 (29, 80) | 40 (30, 64) | 44 (28, 76) | 45 (28, 71) | 46 (30, 76) | 37 (26, 68) | 42 (27, 64) | 47 (28, 72) |
| Tetralogy absent pulmonary valve | 168 (51, 268) | - | - | - | - | - | - | - | - |
| Tetralogy with DORV | 71 (42, 109) | - | 70 (38, 130) | - | 71 (49, 150) | 59 (33, 119) | 68 (34, 116) | 48 (36, 78) | 146 (95, 162) |
| Standard tetralogy | 42 (26, 64) | 44 (25, 74) | 38 (27, 55) | 40 (26, 59) | 41 (28, 65) | 44 (29, 63) | 33 (24, 59) | 40 (26, 63) | 42 (26, 64) |
| **AOS (Total)** | 25 (13, 44) | - | 25 (17, 63) | 20 (16, 35) | 28 (15, 56) | 26 (14, 40) | 23 (11, 48) | 22 (13, 34) | 22 (16, 32) |
| AOS with muti-level left heart obstruction | 38 (26, 68) | - | - | - | 80 (38, 104) | 36 (27, 52) | 42 (22, 51) | 45 (22, 82) | - |
| Isolated AOS | 20 (11, 32) | - | 23 (14, 29) | 17 (15, 26) | 22 (12, 40) | 22 (12, 32) | 17 (10, 38) | 20 (12, 30) | 18 (14, 25) |
| **COA (Total)** | 29 (20, 48) | 38 (28, 51) | 36 (24, 68) | 33 (23, 56) | 33 (23, 58) | 30 (20, 52) | 31 (22, 45) | 29 (19, 48) | 31 (20, 50) |
| COA with VSD | 34 (24, 59) | 45 (38, 63) | 66 (37, 113) | 48 (32, 70) | 47 (30, 74) | 38 (25, 58) | 36 (24, 63) | 33 (22, 57) | 34 (24, 72) |
| Isolated COA | 26 (19, 42) | 30 (23, 46) | 26 (20, 43) | 30 (18, 44) | 28 (20, 44) | 25 (18, 44) | 27 (21, 37) | 24 (18, 39) | 29 (20, 47) |
| **VSD (Total)** | 41 (26, 67) | 47 (25, 86) | 46 (27, 81) | 41 (24, 72) | 44 (26, 84) | 40 (27, 69) | 41 (26, 73) | 41 (26, 66) | 41 (24, 63) |
| Multiple VSDs | 41 (29, 92) | - | 68 (39, 96) | - | 82 (40, 171) | 36 (27, 59) | 71 (44, 196) | 36 (30, 84) | 26 (16, 50) |
| Single VSD | 41 (26, 66) | 48 (25, 84) | 42 (27, 80) | 38 (24, 69) | 42 (25, 74) | 40 (27, 70) | 41 (26, 71) | 41 (26, 65) | 41 (24, 64) |

## Supplementary Table S14: Length of inpatient hospital stay before the age of 1 year by CHD diagnosis subgroup and social determinants (ethnicity and deprivation)

Data are median days (IQR). Patients with missing data ethnicity (n=65, 1.3% of the whole cohort) and deprivation (n=25, 0.6% of the whole cohort) were not included due to limited sample size among each CHD diagnosis.

AOS=congenital aortic stenosis; AVSD=atrioventricular septal defect; COA= coarctation of the aorta; DORV=double outlet right ventricle; FUH=functionally univentricular heart; HLHS=hypoplastic left heart syndrome; TGA=transposition of the great arteries; TOF= tetralogy of Fallot; PA= pulmonary atresia; PS=pulmonary stenosis; VSD=ventricular septal defect.

-Results are not shown (sample size was less than 10).

| **Length of inpatient hospital stay (measured in days) before the age of 1 year** | | | | | | | | | |
| --- | --- | --- | --- | --- | --- | --- | --- | --- | --- |
| **Diagnosis** | **Ethnicity** | | | | **Deprivation** | | | | |
|  | **White** | **Black** | **Asian** | **Mixed / Other** | **Quintile 1 (most deprived)** | **Quintile 2** | **Quintile 3** | **Quintile 4** | **Quintile 5 (least deprived)** |
| **HLHS** | 81 (42, 132) | 108 (43, 224) | 106 (55, 153) | 96 (62, 154) | 97 (46, 150) | 65 (40, 128) | 94 (47, 161) | 93 (52, 138) | 74 (59, 104) |
| **FUH (total)** | 51 (38, 90) | 154 (109, 172) | 50 (29, 70) | 41 (34, 52) | 46 (33, 76) | 53 (43, 96) | 61 (36, 104) | 52 (37, 79) | 48 (38, 84) |
| Double inlet ventricle | 50 (38, 82) | - | 57 (30, 69) | - | 47 (30, 75) | 62 (44, 105) | 64 (38, 117) | - | - |
| Tricuspid atresia | 52 (38, 93) | - | 45 (26, 71) | 38 (31, 44) | 45 (34, 78) | 52 (40, 89) | 42 (33, 88) | 60 (47, 90) | - |
| **TGA (Total)** | 24 (18, 36) | 29 (24, 66) | 30 (21, 56) | 22 (16, 34) | 26 (20, 42) | 28 (18, 48) | 23 (17, 34) | 22 (19, 30) | 26 (19, 38) |
| Complex TGA with PS | 46 (27, 86) | - | 65 (48, 80) | - | - | 65 (33, 88) | 52 (33, 78) | 26 (23, 41) | 44 (19, 78) |
| Complex TGA without PS | 28 (20, 43) | 32 (27, 56) | 46 (30, 66) | 28 (24, 44) | 30 (23, 52) | 29 (20, 54) | 30 (19, 47) | 28 (21, 42) | 27 (20, 36) |
| TGA with intact ventricular septum | 21 (17, 29) | 26 (22, 47) | 23 (19, 30) | 19 (15, 24) | 22 (18, 28) | 24 (18, 33) | 20 (16, 27) | 20 (17, 26) | 24 (18, 32) |
| **PA (Total)** | 48 (28, 89) | 47 (26, 66) | 60 (35, 122) | 38 (32, 86) | 57 (32, 91) | 51 (28, 94) | 48 (28, 104) | 42 (23, 94) | 41 (26, 64) |
| PA with VSD | 62 (32, 115) | - | 61 (36, 130) | 48 (32, 86) | 61 (36, 115) | 66 (32, 109) | 54 (30, 110) | 56 (34, 148) | 52 (34, 104) |
| PA with intact ventricular septum | 34 (23, 54) | - | 55 (28, 99) | - | 43 (29, 59) | 36 (26, 60) | 44 (23, 69) | 20 (17, 31) | 30 (16, 46) |
| **AVSD (Total)** | 42 (21, 74) | 51 (22, 96) | 63 (33, 123) | 44 (20, 74) | 47 (26, 96) | 47 (22, 80) | 48 (26, 84) | 34 (17, 60) | 36 (17, 62) |
| Tetralogy AVSD | 38 (13, 69) | - | 59 (34, 64) | - | 56 (28, 144) | 48 (14, 69) | - | - | - |
| Unbalanced AVSD | 52 (32, 96) | - | - | - | 46 (31, 80) | - | 84 (70, 107) | - | - |
| Complete AVSD | 41 (21, 71) | 48 (22, 97) | 70 (32, 124) | 45 (21, 73) | 47 (26, 98) | 46 (22, 90) | 42 (24, 80) | 32 (17, 63) | 32 (17, 60) |
| **TOF (Total)** | 21 (13, 43) | 30 (16, 56) | 22 (15, 38) | 22 (13, 58) | 25 (15, 47) | 21 (15, 48) | 18 (11, 40) | 21 (13, 40) | 21 (13, 48) |
| Tetralogy absent pulmonary valve | 143 (30, 264) | - | - | - | - | - | - | - | - |
| Tetralogy with DORV | 38 (22, 73) | - | 32 (22, 112) | - | 36 (25, 114) | 35 (20, 78) | 36 (23, 71) | 34 (19, 48) | - |
| Standard tetralogy | 20 (13, 39) | 26 (15, 52) | 20 (13, 32) | 22 (12, 41) | 23 (14, 39) | 20 (15, 38) | 17 (11, 35) | 20 (12, 36) | 20 (13, 41) |
| **AOS (Total)** | 13 (6, 28) | - | 14 (8, 58) | 14 (10, 28) | 16 (6, 38) | 16 (6, 26) | 14 (7, 34) | 11 (7, 26) | 10 (8, 15) |
| AOS with muti-level left heart obstruction | 26 (15, 42) | - | - | - | 33 (26, 95) | 25 (20, 38) | 24 (14, 41) | 22 (10, 52) | - |
| Isolated AOS | 9 (5, 21) | - | 9 (4, 22) | 10 (10, 16) | 10 (4, 26) | 12 (5, 18) | 10 (5, 20) | 10 (6, 17) | 9 (6, 10) |
| **COA (Total)** | 19 (13, 34) | 25 (16, 43) | 24 (18, 46) | 26 (14, 43) | 24 (15, 42) | 20 (13, 33) | 21 (14, 32) | 18 (12, 32) | 20 (12, 35) |
| COA with VSD | 25 (17, 42) | 35 (21, 56) | 40 (25, 94) | 36 (24, 55) | 36 (23, 59) | 26 (17, 40) | 25 (18, 45) | 23 (17, 43) | 24 (14, 40) |
| Isolated COA | 17 (12, 29) | 23 (16, 34) | 19 (13, 28) | 23 (12, 32) | 19 (13, 32) | 16 (12, 30) | 17 (12, 24) | 15 (10, 26) | 18 (11, 30) |
| **VSD (Total)** | 22 (12, 46) | 32 (14, 66) | 28 (15, 58) | 21 (10, 49) | 27 (14, 57) | 22 (12, 45) | 23 (12, 50) | 22 (12, 44) | 18 (9, 43) |
| Multiple VSDs | 25 (14, 62) | - | 48 (23, 64) | - | 54 (22, 151) | 22 (10, 40) | 42 (12, 168) | 25 (22, 67) | 24 (12, 37) |
| Single VSD | 21 (11, 46) | 32 (14, 64) | 26 (15, 54) | 21 (10, 46) | 25 (13, 52) | 22 (12, 46) | 23 (12, 47) | 21 (11, 44) | 17 (10, 43) |

## Supplementary Table S15: Length of outpatient hospital stay before the age of 1 year by CHD diagnosis subgroup and social determinants (ethnicity and deprivation)

Data are median days (IQR). Patients from Wales (n=235, 4.8% of the whole cohort) were not included because we don’t have their outpatient records. Additionally, patients with missing data ethnicity (n=10) and deprivation (n=25) were not included due to limited sample size among each CHD diagnosis.

AOS=congenital aortic stenosis; AVSD=atrioventricular septal defect; COA= coarctation of the aorta; DORV=double outlet right ventricle; FUH=functionally univentricular heart; HLHS=hypoplastic left heart syndrome; TGA=transposition of the great arteries; TOF= tetralogy of Fallot; PA= pulmonary atresia; PS=pulmonary stenosis; VSD=ventricular septal defect.

-Results are not shown (sample size was less than 10).

| **Length of hospital stay (measured in days; including inpatient, outpatient and emergency care services) before the age of 1 year** | | | | | | | | | |
| --- | --- | --- | --- | --- | --- | --- | --- | --- | --- |
| **Diagnosis** | **Ethnicity** | | | | **Deprivation** | | | | |
|  | **White** | **Black** | **Asian** | **Mixed / Other** | **Quintile 1 (most deprived)** | **Quintile 2** | **Quintile 3** | **Quintile 4** | **Quintile 5 (least deprived)** |
| **HLHS** | 12 (1, 23) | 11 (4, 22) | 7 (0, 19) | 12 (6, 24) | 9 (0, 17) | 12 (0, 25) | 14 (7, 20) | 20 (8, 29) | 10 (1, 30) |
| **FUH (total)** | 20 (12, 30) | 14 (6, 26) | 20 (11, 32) | 15 (11, 20) | 15 (10, 26) | 19 (12, 32) | 20 (10, 28) | 22 (15, 29) | 19 (13, 47) |
| Double inlet ventricle | 20 (12, 29) | - | 21 (9, 32) | - | 17 (10, 27) | 20 (13, 30) | 20 (12, 35) | - | - |
| Tricuspid atresia | 19 (10, 30) | - | 18 (12, 31) | 15 (15, 20) | 15 (12, 24) | 18 (13, 35) | 21 (10, 24) | 20 (14, 29) | - |
| **TGA (Total)** | 7 (4, 11) | 7 (4, 12) | 8 (4, 14) | 5 (3, 8) | 6 (3, 11) | 6 (4, 11) | 7 (4, 10) | 6 (4, 12) | 8 (5, 13) |
| Complex TGA with PS | 13 (6, 19) | - | 18 (14, 47) | - | 11 (7, 13) | 16 (11, 36) | 12 (2, 30) | 12 (5, 16) | 20 (14, 35) |
| Complex TGA without PS | 8 (4, 13) | 7 (6, 10) | 8 (5, 14) | 8 (6, 10) | 7 (4, 13) | 8 (5, 12) | 8 (6, 18) | 9 (5, 14) | 8 (5, 10) |
| TGA with intact ventricular septum | 6 (4, 9) | 5 (3, 9) | 5 (4, 9) | 4 (3, 6) | 5 (3, 9) | 5 (4, 8) | 6 (4, 9) | 5 (4, 8) | 6 (5, 13) |
| **PA (Total)** | 14 (9, 26) | 18 (12, 27) | 18 (9, 29) | 18 (10, 23) | 13 (8, 22) | 18 (11, 33) | 15 (9, 23) | 18 (12, 40) | 15 (8, 27) |
| PA with VSD | 14 (10, 27) | - | 19 (13, 29) | 20 (13, 26) | 14 (9, 22) | 21 (12, 40) | 15 (10, 24) | 19 (12, 30) | 14 (8, 28) |
| PA with intact ventricular septum | 15 (7, 19) | - | 11 (4, 24) | - | 12 (5, 17) | 17 (10, 22) | 12 (6, 16) | - | 16 (10, 21) |
| **AVSD (Total)** | 18 (11, 27) | 16 (10, 23) | 17 (5, 25) | 14 (8, 22) | 14 (9, 21) | 18 (11, 29) | 21 (14, 34) | 19 (12, 31) | 18 (11, 28) |
| Tetralogy AVSD | 39 (14, 48) | - | 17 (13, 20) | - | 16 (12, 20) | 21 (17, 57) | - | - | - |
| Unbalanced AVSD | 19 (10, 26) | - | - | - | 19 (9, 26) | - | 21 (19, 24) | - | - |
| Complete AVSD | 17 (11, 26) | 16 (10, 23) | 17 (4, 24) | 14 (9, 22) | 13 (8, 19) | 18 (11, 28) | 19 (13, 31) | 19 (12, 31) | 18 (10, 26) |
| **TOF (Total)** | 15 (10, 25) | 11 (6, 18) | 15 (8, 22) | 13 (7, 18) | 14 (9, 22) | 14 (9, 26) | 14 (9, 20) | 16 (10, 24) | 16 (11, 25) |
| Tetralogy absent pulmonary valve | 11 (6, 23) | - | - | - | - | - | - | - | - |
| Tetralogy with DORV | 17 (12, 33) | - | 22 (13, 34) | - | 20 (12, 26) | 15 (7, 33) | 15 (13, 30) | 16 (11, 29) | 34 (15, 44) |
| Standard tetralogy | 14 (10, 23) | 11 (6, 18) | 15 (8, 21) | 13 (9, 19) | 13 (9, 21) | 14 (10, 26) | 14 (9, 20) | 16 (10, 25) | 16 (10, 23) |
| **AOS (Total)** | 8 (4, 13) | - | 6 (4, 9) | 6 (5, 9) | 8 (4, 11) | 8 (5, 13) | 7 (4, 11) | 7 (5, 10) | 9 (7, 14) |
| AOS with muti-level left heart obstruction | 8 (6, 13) | - | - | - | 9 (4, 12) | 10 (7, 13) | 8 (5, 10) | 10 (6, 24) | - |
| Isolated AOS | 7 (4, 12) | - | 6 (5, 8) | 6 (4, 7) | 7 (4, 10) | 7 (5, 12) | 6 (3, 10) | 6 (4, 10) | 9 (7, 13) |
| **COA (Total)** | 7 (4, 12) | 8 (5, 14) | 8 (5, 13) | 5 (4, 10) | 7 (4, 13) | 7 (5, 12) | 7 (5, 11) | 7 (4, 12) | 8 (5, 14) |
| COA with VSD | 8 (5, 12) | 9 (5, 16) | 13 (8, 21) | 6 (4, 11) | 8 (4, 13) | 8 (6, 15) | 8 (5, 12) | 8 (4, 12) | 9 (4, 13) |
| Isolated COA | 7 (4, 12) | 7 (5, 13) | 7 (5, 9) | 5 (5, 7) | 7 (4, 11) | 6 (4, 10) | 7 (4, 10) | 6 (4, 12) | 8 (5, 14) |
| **VSD (Total)** | 12 (7, 21) | 12 (9, 17) | 13 (8, 20) | 12 (7, 18) | 12 (7, 18) | 12 (7, 19) | 12 (7, 21) | 13 (9, 22) | 14 (9, 22) |
| Multiple VSDs | 12 (7, 22) | - | 13 (8, 21) | - | 15 (8, 22) | 9 (6, 16) | 12 (6, 22) | 12 (7, 22) | 7 (5, 13) |
| Single VSD | 12 (8, 20) | 12 (9, 17) | 13 (8, 20) | 12 (7, 18) | 12 (7, 18) | 12 (7, 19) | 12 (7, 20) | 13 (9, 22) | 15 (10, 22) |
